# Sensitizing tumor response to topoisomerase I antibody-drug conjugate by selective CDK7 inhibition

**DOI:** 10.1126/sciadv.aeb4023

**Published:** 2026-09-30

**Authors:** Hyerim Jung, Yoonji Lee, Donghoon Yu, Yeejin Jeon, Hwankyu Kang, Dahun Um, Mooyoung Seo, Dongsik Park, Jeongjun Kim, Seung-Joo Lee, Jaeseung Kim, Lauren Escobedo, Yilun Sun, Anish Thomas, Kiyean Nam, Tae-Kyung Kim

**Affiliations:** ^1^ Department of Life Sciences, Pohang University of Science and Technology, Pohang, Gyeongbuk 37673, Korea.; ^2^ Qurient Co. Ltd., C801, 242 Pangyo-ro, Bundang-gu, Seongnam-si, Gyeonggi-do 13487, Korea.; ^3^ University of Maryland Marlene & Stewart Greenebaum Comprehensive Cancer Center, University of Maryland School of Medicine, Baltimore, MD 21201, USA.; ^4^ Developmental Therapeutics Branch, Center for Cancer Research, National Cancer Institute, NIH, Bethesda, MD 20892, USA.

## Abstract

This study investigates the transcriptional impact of Q901, a highly selective cyclin-dependent kinase 7 (CDK7) inhibitor in clinical development. Q901 primarily disrupted MYC and E2F-dependent transcription programs, down-regulating cell cycle control and DNA damage repair pathways. CDK7 binding at the promoter-proximal regions was markedly stabilized by Q901, leading to reduced occupancy of MYC, E2F, and RNA polymerase II (RNAPII). These findings offered a novel therapeutic strategy to enhance cancer susceptibility to topoisomerase I (TOP1) DNA-protein cross-links (TOP1-DPCs) induced by TOP1 inhibitors. Resistance to TOP1 inhibitors arises through activation of DNA repair pathways when elongating RNAPII encounters TOP1-DPCs. By suppressing RNAPII transition from initiation to elongation and DNA repair pathways, Q901 stabilizes TOP1-DPCs and sensitizes tumors to TOP1 inhibitors. Preclinical studies demonstrated enhanced tumor suppression when combining Q901 with TOP1 inhibitor–based antibody-drug conjugates (TOP1i-ADCs), highlighting its potential as a therapeutic option for cancers resistant to TOP1i-ADC therapy.

## INTRODUCTION

Cyclin-dependent kinase 7 (CDK7), as part of the CDK-activating kinase (CAK) complex (comprising CDK7, cyclin H, and MAT1), plays a pivotal role in regulating both cell cycle and transcription (*1*, *2*). The CAK complex facilitates the phosphorylation of the T-loop in CDK1, CDK2, CDK4, and CDK6, which is essential for their activation leading to cell cycle progression (*3*). In transcription, CDK7 functions as a core component of the transcription factor II H (TFIIH) complex, where it plays critical roles in both the initiation and elongation phases of RNA polymerase II (RNAPII)–mediated transcription (*4*–*7*). During initiation, CDK7 phosphorylates the Ser^5^ residue of the RNAPII C-terminal domain (CTD), a process that facilitates promoter escape (*4*, *5*). In the elongation phase, CDK7 phosphorylates CDK9, which, in turn, phosphorylates the pausing factors NELF and DSIF, as well as the RNAPII CTD at the Ser^2^ residue (*6*). This phosphorylation leads to the release of NELF, the conversion of DSIF into a positive elongation factor, and the continuation of transcription elongation (*7*). In addition, these phosphorylation events are crucial for recruiting capping enzymes and splicing factors, which regulate RNA processing (*8*). Thus, CDK7-mediated phosphorylation of RNAPII is integral to the regulation of transcription and RNA processing. The dual role of CDK7 in cell cycle regulation and transcription underscores its importance as a fundamental regulator of cellular processes.

Cancer is characterized by complex molecular alterations, including changes in gene expression mediated by TFs, chromatin remodelers, or histone-modifying enzymes (*9*). Targeting each of these factors individually is challenging due to their complexity and sheer number. Instead, the general transcriptional machinery, which is essential for gene transcription, has emerged as a viable therapeutic target. CDK7, as a key component of this machinery, presents an attractive target for cancer therapy. CDK7 expression is often elevated in many types of cancer with poor prognosis (*10*, *11*). Previously developed CDK7 inhibitors, such as THZ1 and SY-1365, have demonstrated anticancer effects by suppressing RNAPII CTD phosphorylation and inducing global transcriptional down-regulation. However, their cytotoxic effects were attributed to off-target activity on CDK12 and CDK13 (*12*, *13*). In contrast, a newer inhibitor YKL-5-124, which exhibits greater specificity for CDK7 without affecting other CDKs, did not reduce RNAPII CTD phosphorylation or cause global transcriptional down-regulation (*14*). THZ1 and its derivatives, SY-1365 and SY-5609, were shown to arrest the cell cycle at the G_2_-M phase and induce apoptosis (*12*, *13*, *15*), whereas YKL-5-124 primarily induced G_1_ cell cycle arrest without noticeable apoptosis (*14*). Similarly, genetic disruption of CDK7 did not result in global transcriptional down-regulation or inhibition of CTD phosphorylation (*16*).

Topoisomerase I (TOP1) is a critical enzyme that alleviates torsional stress in DNA strands during replication and transcription by transiently introducing single-strand breaks into DNA. During this process, TOP1 forms a covalent bond between its catalytic tyrosine (Tyr) residue and the 3′ end of the broken DNA backbone, generating transient and reversible TOP1 cleavage complexes (TOP1ccs). TOP1 inhibitors, such as topotecan (TPT) and irinotecan, stabilize TOP1ccs and convert them into irreversible TOP1 DNA-protein cross-links (TOP1-DPCs), thereby blocking the progression of transcription and replication forks, ultimately leading to DNA damage and subsequent cell death (*17*, *18*). TOP1 inhibitors are widely used as payloads in antibody-drug conjugates (ADCs) (*19*), a therapeutic approach that combines the specificity of monoclonal antibodies with the cytotoxic effects of small-molecule drugs (*20*). Trastuzumab deruxtecan (T-DXd), a Food and Drug Administration (FDA)–approved TOP1 inhibitor–based ADC (TOP1i-ADC) showed an impressive objective response rate (ORR) of 60.9% and median progression-free survival (PFS) of 16.4 months in patients with HER2-positive metastatic breast cancer who had been previously treated with multiple lines of HER2-targeted therapies (*21*). More recently, T-DXd has shown notable survival benefits in HER2-low metastatic breast cancer, expanding its use beyond the HER2-positive patient population (*22*). To further enhance the efficacy of TOP1i-ADCs, drug combination strategies are also being actively explored. A combination of TOP1i-ADC with either a DNA damage response inhibitor (e.g., PARP or ATR inhibitor) or immunotherapy (e.g., anti–PD-1 antibody) has been shown to increase the anticancer efficacy of TOP1i-ADC (*23*, *24*).

Theoretically, cancer cells with high transcription and replication rates are expected to be more susceptible to TOP1 inhibitors. However, a potential limitation of TOP1 inhibitor therapy lies in the ability of cells to repair DNA damage through transcription-coupled repair (TCR). Studies have shown that collisions between elongating RNAPII and TOP1-DPCs can activate the ubiquitin-proteasome pathway, leading to the degradation of both complexes, followed by DNA repair (*18*). A recent study revealed that the sensitivity of rapidly replicating small cell lung cancer (SCLC) cells to TPT can be enhanced when combined with the CDK7 inhibitor, THZ1. Mechanistically, targeting transcription with THZ1 reduces collisions between RNAPII and TOP1-DPCs, thereby preventing the proteasomal degradation of TOP1-DPCs (*25*). This combination represents a novel strategy to increase the efficacy of TOP1 inhibitors, particularly in the context of balancing the therapeutic efficacy and toxicity of TOP1i-ADCs. However, a deeper understanding of the mechanism of action is needed to successfully translate the combination of CDK7 inhibitors and TOP1i-ADCs into clinical applications.

The present study explores the molecular mechanism of CDK7 inhibition in transcriptional regulation using Q901 (mocaciclib), a novel and highly selective CDK7 inhibitor. Q901 engages the adenosine triphosphate (ATP)–binding pocket of CDK7 while forming a covalent bond with Cys^312^, a residue located outside the ATP-binding pocket. This covalent anchoring stabilizes the compound within the ATP-binding site, thereby enhancing inhibition of CDK activity in cells. By selectively inhibiting CDK7, Q901 traps CDK7 and disrupts the RNAPII transcription process at promoter-proximal regions of genes involved in MYC- and E2F-driven oncogenic transcription programs. Q901 prevents RNAPII from transitioning into stable elongation, leading to the stabilization of TOP1-DPCs, which provides a potential mechanism for overcoming cancer resistance to TOP1 inhibitors. Preclinical studies further demonstrated that Q901 substantially improves the therapeutic effect of TOP1i-ADCs, resulting in enhanced tumor suppression. These findings underscore Q901’s potential as a promising therapeutic option, particularly for cancers resistant to TOP1i-ADC therapies.

## RESULTS

### Q901 is a highly selective covalent inhibitor of CDK7

Q901 is a covalent ATP-competitive inhibitor featuring a pyrazolo[1,5-*a*][1,3,5]triazine as a hinge-binding motif and an α-fluoroacrylamide as its covalent warhead (Fig. 1A and fig. S1A). It engages the ATP-binding pocket in a competitive manner while forming a covalent bond with Cys^312^—a residue located outside the ATP-binding pocket in the C-terminal region of CDK7—thereby anchoring the compound and enhancing inhibition. Mass spectrometry analysis demonstrated that the intensity of the cysteine-containing peptide PNCPVETLKEQSNPALAIKR (residues 310 to 329) and PNCPVETLK (residues 310 to 318) was reduced by ∼99% following Q901 treatment. In contrast, the intensity of another cysteine-containing peptide, PGPTPGCQLPR (residues 299 to 309) and RPGPTPGCQLPR (residues 298 to 309), remained unaffected, indicating a site-specific interaction between Q901 and Cys^312^ (Fig. 1B and fig. S1B).

**Fig. 1.**
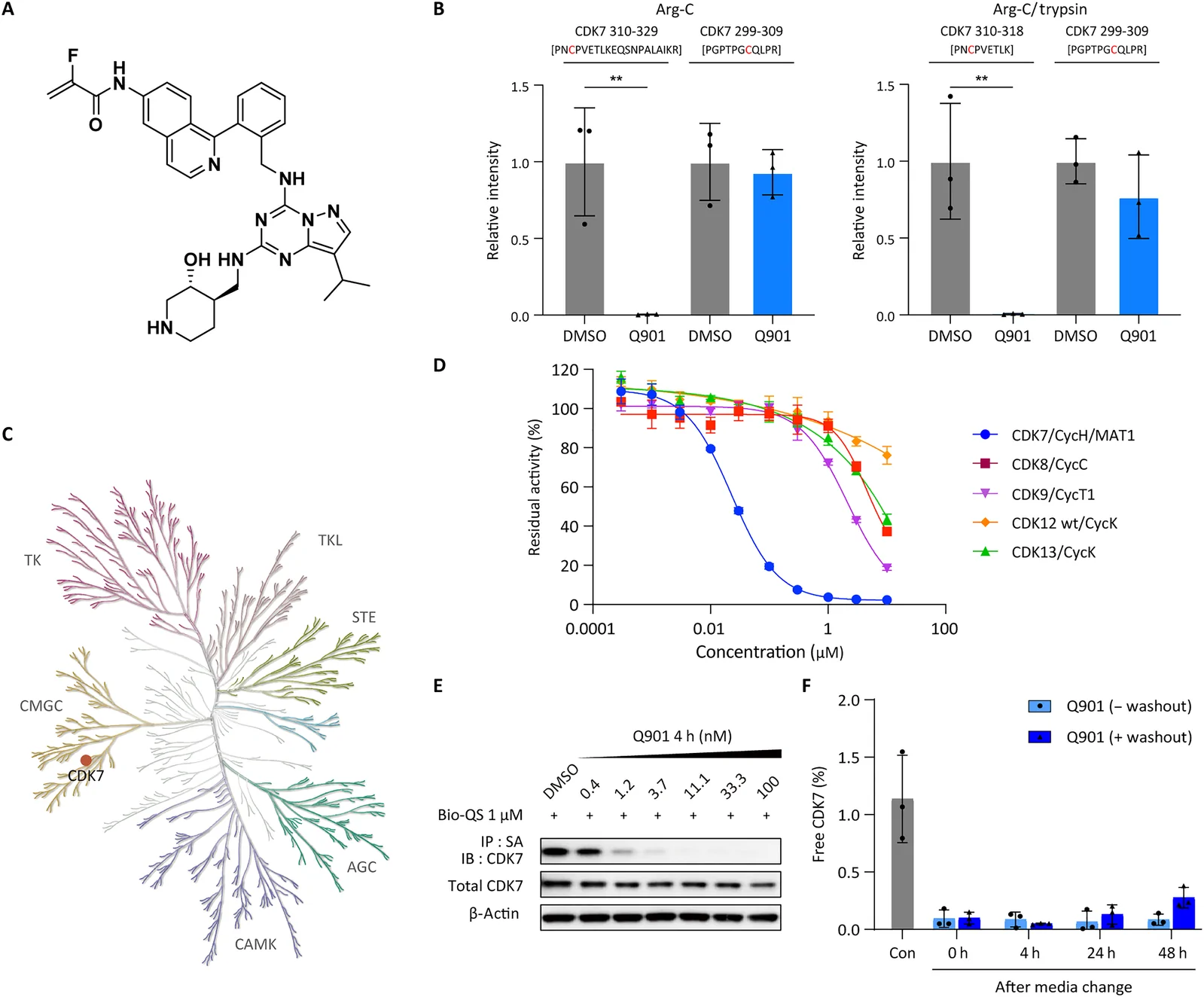
Q901 is a CDK7 selective covalent inhibitor. (**A**) Chemical structure of Q901. (**B**) Covalent binding site analysis by mass spectrometry analysis of the recombinant CDK7/cyclin H/MAT1 (CAK) trimeric complex. The recombinant CAK complex was incubated with Q901 or DMSO for 1 hour, digested for 18 hours, and analyzed by mass spectrometry (*n* = 3; unpaired *t* test with Welch’s correction, data represent means ± SD, **P ≤ 0.01.). (**C**) KinMap image illustrating the kinase inhibition profile of Q901 against a panel of 410 kinases (397 protein kinase assays and 13 lipid kinase assays). Residual activity was measured at 1 μM for 1 hour using the PanQinase assay, with ATP concentrations set at the apparent ATP-*K*_m_ for each kinase. CDK7 showed 99% inhibition. (**D**) Efficacy and selectivity of Q901 against other CDKs at ATP concentrations corresponding to the apparent ATP-*K*_m_ value for each kinase. Residual activity (%) was measured after a 1-hour incubation at the indicated concentrations (*n* = 3; two-way ANOVA with Tukey’s multiple comparisons test, data represent means ± SD). (**E**) CDK7 target occupancy assay using Bio-QS, a biotinylated analog of Q901. A2780 cells were treated with Q901 or DMSO for 4 hours (h) and subjected to IP using Bio-QS and streptavidin agarose beads, followed by immunoblotting for CDK7. (**F**) Washout-based target occupancy assay to measure the duration of CDK7 inhibition. A2780 cells treated with 6 nM Q901 for 4 hours were washed with fresh medium and incubated for the indicated times. Cells were lysed, treated with Bio-QS, and immunoprecipitated with streptavidin agarose beads (SA). The percentage of free CDK7 was calculated by normalizing CDK7 levels in IP samples from Q901 treatment to those in IP samples from the DMSO-treated group (*n* = 3; two-way ANOVA with Tukey’s multiple comparisons test, data represent means ± SD).

To evaluate whether the anticancer efficacy of Q901 depends on covalent engagement at Cys^312^, we compared Q901 with two structurally related noncovalent analogs: QS0551546 (Q901R), which differs from Q901 by a single hydrogen atom in the α-fluoroacrylamide warhead and thus adopts the same binding mode but cannot form a covalent bond, and QS0551508, which lacks the entire covalent binding unit while retaining similar CDK7 binding affinity (figs. S2, A and B, and S3A). In cell-free enzymatic assays, all three compounds exhibited comparable inhibitory potencies against CDK7: Q901 [median inhibitory concentration (IC_50_) = 10.37 nM], QS0551508 (IC_50_ = 5.51 nM), and Q901R (IC_50_ = 10.02 nM), indicating that reversible binding to the active site is sufficient for enzymatic inhibition (fig. S3B). However, in cellular proliferation assays, Q901 displayed markedly greater antiproliferative activity than the noncovalent analogs across multiple cancer cell lines (fig. S3C). These results indicate that covalent modification of Cys^312^, although dispensable for enzymatic inhibition in vitro, is critical for sustained CDK7 inhibition and potent cellular cytotoxicity, thereby distinguishing Q901 as a promising drug candidate.

In vitro, Q901 inhibited CDK7 activity with an IC_50_ of 10 nM but exhibited much lower inhibitory activity against other kinases in an inhibition profiling assay with a panel of 410 kinases, including 13 lipid kinases (Fig. 1, C and D; fig. S4A; and table S1). The inhibition by Q901 was highly selective for CDK7, demonstrating a residual activity value of 1 (99% inhibition) at a concentration of 1 μM. In comparison, the IC_50_ values for other CDKs were substantially higher than that for CDK7, ranging from 242-fold to over 1000-fold (table S1). In addition, Q901 binding to CDK7 remained stable even after washout in a target occupancy assay, consistent with its covalent binding activity (Fig. 1, E and F, and fig. S4B).

Special attention was given to the selectivity of Q901 against CDK8, CDK9, CDK12, and CDK13 as these CDKs are also involved in transcriptional regulation. Inhibitory activities against these CDKs could confound the assessment of Q901’s effects on transcription via CDK7 inhibition, as noted in prior studies with other CDK7 inhibitors such as THZ1 (*12*–*14*). The IC_50_ values of Q901 were found to be greater than 10 μM for CDK8, 8.58 μM for CDK9, 2.48 μM for CDK12, and 7.69 μM for CDK13. The fold differences in Q901-mediated inhibition of these CDKs compared to CDK7 were >1000 for CDK8, 715 for CDK9, 207 for CDK12, and 641 for CDK13 (Fig. 1D and table S1). These analyses collectively highlight the superior selectivity and efficacy of Q901 as a CDK7 inhibitor compared to previously characterized inhibitors (*12*–*14*).

### Q901 alters RNAPII transcription profiles

Various cancer types, including estrogen receptor–positive (ER+) breast cancer, exhibit elevated CDK7 expression compared with normal tissues, which is associated with poorer patient outcomes. (*9*). MCF-7 cells, a representative ER-positive breast cancer model, also exhibit high expression levels of CDK7 (*11*). Inhibition of CDK7 has been shown to suppress cell cycle progression and induce apoptosis in MCF-7 cells, suggesting that targeting CDK7 could be a viable therapeutic strategy for breast cancer (*26*). For this reason, we used ER-positive MCF-7 cells as a model to evaluate Q901 efficacy. Q901 effectively inhibited MCF-7 cell growth, with IC_50_ values of 95 nM at 72 hours, 16 nM at 96 hours, and 5 nM at 120 hours (Fig. 2A).

**Fig. 2.**
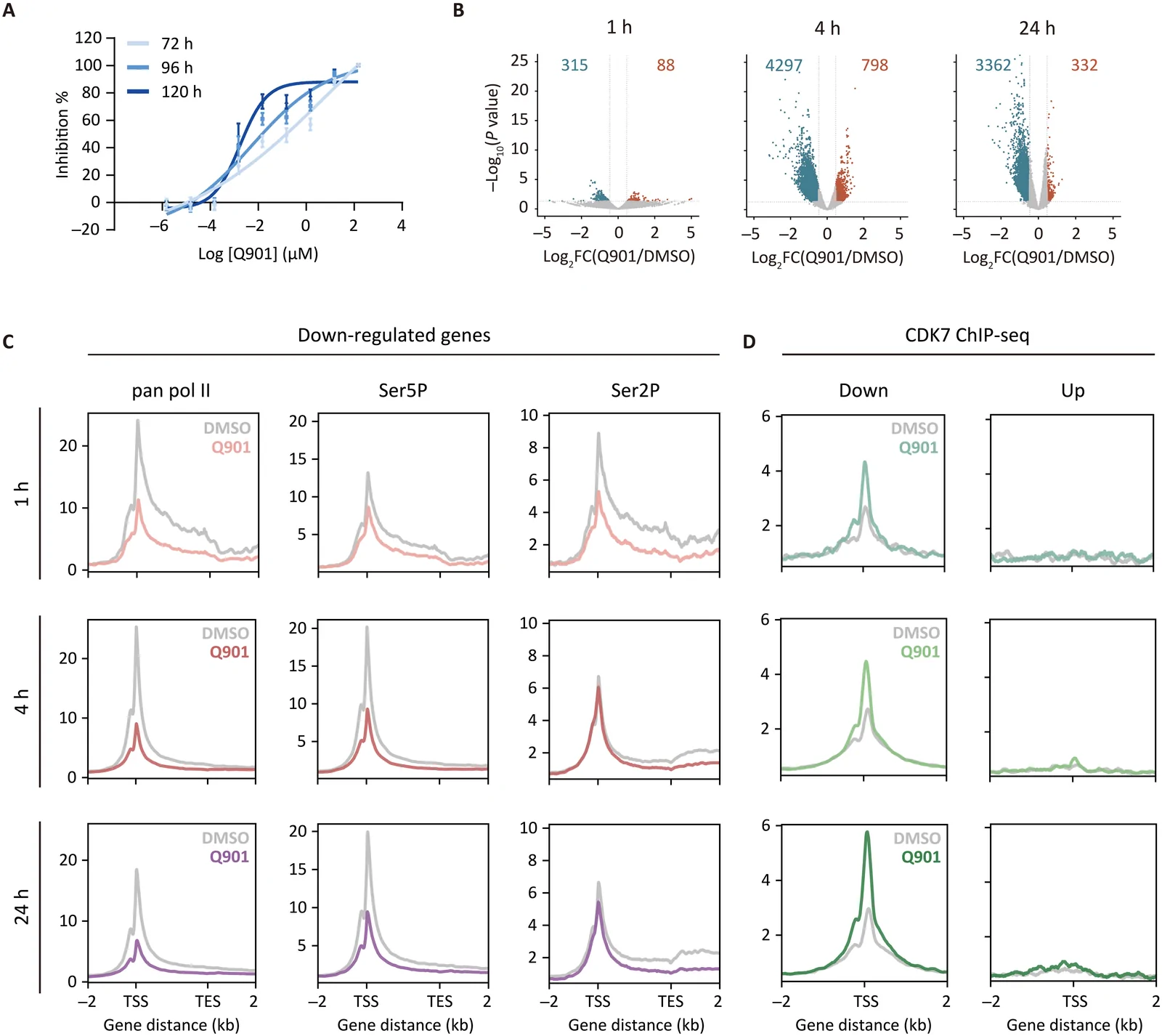
Q901 alters RNAPII phosphorylation and transcriptional activity. (**A**) MCF-7 cells were treated with Q901 at the indicated concentrations for 72, 96, or 120 hours. Cell viability was measured using the ATP Lite system. Inhibition (%) was plotted against the log-transformed Q901 concentration (μM) (*n* = 3 to 4). Data represent means ± SD. (**B** and **C**) RNAPII ChIP-seq was performed following treatment with 100 nM Q901 for the indicated duration. (B) Volcano plot of pan RNAPII ChIP-seq signals after Q901 treatment [*n* = 2; blue dot indicates *P* value ≤ 0.05 and log_2_ fold change (log_2_FC) ≤ −0.58; red dot indicates *P* value ≤ 0.05 and log_2_FC ≥ 0.58]. (C) Average ChIP-seq signal plots of various RNAPII forms for genes down-regulated by Q901. (**D**) Average CDK7 ChIP-seq signal plots of down-regulated and up-regulated genes at the TSS. CDK7 ChIP-seq was performed with 100 nM Q901 for the indicated durations.

To assess the phosphorylation levels of the CTD in RNAPII bound to chromatin, chromatin immunoprecipitation sequencing (ChIP-seq) experiments were conducted using specific antibodies targeting individual phosphorylated serine residues (Fig. 2, B and C, and fig. S5A). Volcano plot analysis revealed reduced RNAPII binding in thousands of genes following Q901 treatment, with the magnitude of reduction increasing over time (Fig. 2, B and C, and fig. S5B). Hundreds of genes including the gene encoding the largest subunit of RNAPII (POLR2A), a biomarker for pharmacodynamic (PD) responses to CDK7 inhibitors (*15*, *27*) exhibited a slight increase in RNAPII binding (fig. S5, C and D). However, the average level of pan RNAPII in these up-regulated genes was substantially lower than that in down-regulated genes. In addition, a slight increase in RNAPII levels was observed only at the 1- and 4-hour time points after Q901 treatment but not at 24 hours. This suggests that the observed increase in RNAPII binding may be a temporary effect resulting from the global down-regulation of RNAPII binding to chromatin as part of a compensatory mechanism. In contrast, down-regulated genes exhibited substantially higher levels of basal transcription. Analysis of their mRNA half-lives showed a trend toward a negative correlation between the degree of transcriptional down-regulation and mRNA stability; specifically, log_2_ fold change [Q901/dimethyl sulfoxide (DMSO)] values were relatively higher in mRNAs with shorter half-lives (fig. S5E). This finding aligns with a previous study indicating that CDK7 inhibition in A549 cells primarily down-regulated short-lived genes with high mRNA turnover rates (*2*).

The occupancy levels of Ser^2^ and Ser^5^ phosphorylated forms of RNAPII were also substantially decreased upon Q901 treatment (Fig. 2C and fig. S5A). The decrease in Ser^5^ phosphorylation levels most likely resulted from the direct inhibition of CDK7 by Q901. Unlike the Ser^5^ phosphorylation profiles, which showed strong enrichment near the transcription start sites (TSSs), the decrease in Ser^2^ phosphorylation was more prominent in the gene coding region, especially toward the transcription end sites (TESs) at later time points of Q901 treatment. This result was expected, as CDK9, a primary kinase that phosphorylates Ser^2^ during the elongation phase, needs to be activated by CDK7 through phosphorylation (*6*).

Q901 also altered the binding profile of CDK7 at the promoter regions (Fig. 2D and fig. S5A). TFIIH associates with the promoter to form a preinitiation complex with RNAPII, facilitating the transition from initiation to elongation (promoter clearance). Following this, it is released from early elongation complexes and recycled for subsequent rounds of transcription (*7*). However, Q901 treatment substantially increased CDK7 occupancy over time, indicating that covalently inhibited CDK7 remained stably bound to the promoter without being released (Fig. 2D, left). This phenomenon was not observed in up-regulated genes. No notable CDK7 signals were detected at the promoters of up-regulated genes, regardless of Q901 treatment (Fig. 2D, right). It remains unclear whether the up-regulated genes can circumvent CDK7 action during the transcription cycle or if the observed results are merely due to detection sensitivity issues stemming from the substantially lower levels of basal transcription in this gene group.

To determine whether these results depend on covalent engagement of Q901, we performed parallel ChIP-seq analysis using Q901R, a noncovalent analog. Q901R exhibited markedly attenuated effects: Unlike Q901, it did not substantially increase CDK7 occupancy at promoters except at the 24-hour time point, and promoter-associated RNAPII occupancy was reduced to a much lesser extent (fig. S5F). These results further support that covalent tethering of Q901 to Cys^312^ is a key determinant of its efficacy in disrupting the RNAPII transcription cycle.

Together, our findings support a model in which the inhibition of CDK7 activity alters the dynamics of transcription initiation complexes, as previously described (*2*, *6*, *28*, *29*). Given CDK7’s established role in RNAPII pausing (*6*, *8*, *28*, *29*), the reduced occupancy of RNAPII at promoter-proximal regions upon Q901 treatment likely results from the rapid clearance of RNAPII complexes from their pausing sites. Furthermore, a recent study has shown that CDK7 activity is crucial for the efficient release of transcription initiation factors and the Mediator complex (*28*). In this context, the observed Q901-mediated trapping of CDK7 could lead to the prolongation of the RNAPII preinitiation complex at the promoters, preventing TF cycling.

### Q901 impairs enhancer function

Super-enhancers (SEs) are clusters of enhancers enriched with various TFs, RNAPII machinery, and chromatin regulators that control key identity genes by driving high transcription levels of both enhancer RNAs (eRNAs) and target gene mRNAs (*30*, *31*). Dysregulation of SE activity is common in cancer, creating transcriptional addiction that provides a therapeutic opportunity to target the general transcription machinery (*9*).

To investigate Q901’s effect on SE-driven transcription, we identified 483 SEs and 8134 typical enhancers (TEs) in MCF-7 cells using publicly available H3K27ac ChIP-seq data and the ROSE program (fig. S6A) (*32*). The enhancers were classified into four groups: CDK7-bound SEs, CDK7-nonbound SEs, CDK7-bound TEs, and CDK7-nonbound TEs. Both SEs and TEs were strongly enriched with CDK7 and RNAPII, with SEs exhibiting stronger enrichment than TEs (Fig. 3A). A similar pattern was observed for eRNA profiles using fastGRO (fast Global Run-On), reflecting transcription activity at enhancers (fig. S6B).

**Fig. 3.**
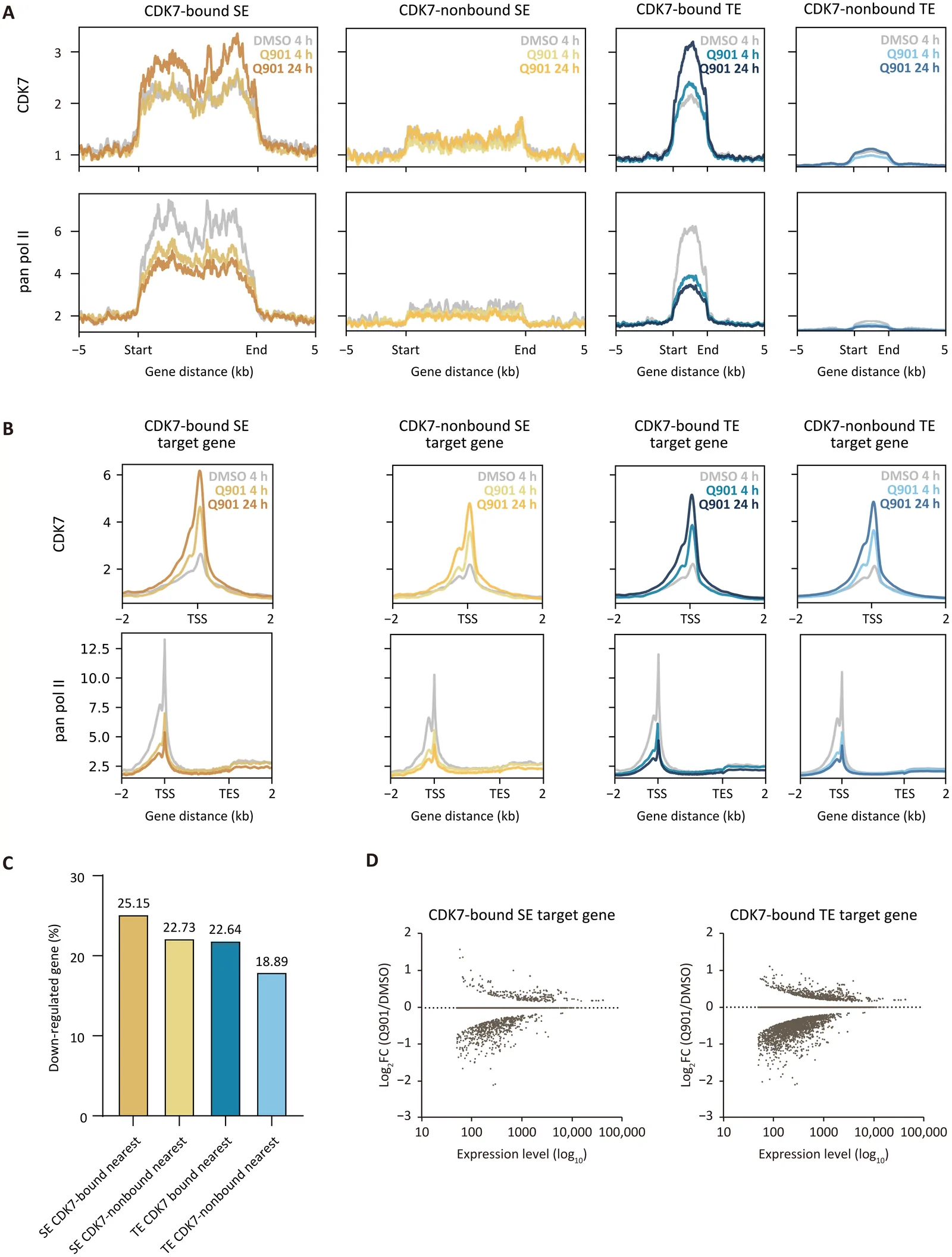
Q901 reduces enhancer-associated transcriptional activity. (**A**) Average ChIP-seq signal plots of CDK7 and pan RNAPII ChIP-seq across four enhancer groups. (**B**) Average ChIP-seq signal plots for CDK7 and pan RNAPII for protein-coding genes regulated by four enhancer groups. (**C**) Bar graph shows the proportion of down-regulated genes in each enhancer groups (pan RNAPII ChIP-seq; *n* = 2; *P* value ≤ 0.05 and log_2_FC ≤ −0.58). (**D**) Scatterplot shows the expression levels and log_2_FC of target genes regulated by CDK7-bound SE and CDK7-bound TE (pan RNAPII ChIP-seq; *n* = 2; *P* value ≤ 0.05).

Q901-mediated inhibition of CDK7 activity substantially reduced RNAPII binding at SEs and TEs, suggesting that Q901 impaired enhancer activities (Fig. 3A and figs. S6, B and C, and S7A). To examine the impact of enhancer inhibition on target gene transcription, RNAPII and CDK7 binding levels were assessed in genes located within ±50 kb from the enhancer regions (Fig. 3B and fig. S7B). Baseline RNAPII binding was highest in the target genes of CDK7-bound SEs (fig. S6D). The proportion of down-regulated genes was also highest in that group (∼25% of potential target genes) (Fig. 3C). However, the degrees of transcriptional alteration caused by Q901 were comparable across the four enhancer target gene groups, with no strong bias toward SE-regulated genes (Fig. 3, C and D).

Notably, Gene Ontology (GO) analysis revealed that genes involved in nucleosome organization were primarily affected by CDK7 inhibition in SEs, whereas the down-regulation of genes related to translation resulted from alterations in TE activities (fig. S6E). These findings suggest that the anticancer effect of Q901 is, in part, mediated by suppressing the transcription of genes necessary for cell growth and proliferation through modulation of enhancer activities.

### CDK7 inhibition impairs key oncogenic pathways

To further elucidate the molecular consequences of CDK7 inhibition, we performed Gene Set Enrichment Analysis (GSEA) using hallmark gene sets (Fig. 4A). Down-regulated genes were substantially enriched in pathways canonically associated with oncogenesis, including cell cycle control, DNA repair mechanisms, and targets of the oncogenic TFs MYC and E2F. CDK7 occupancy at these pathway genes was markedly elevated over time with Q901 treatment, whereas RNAPII levels were reciprocally diminished, substantiating the efficacy of Q901 as a CDK7 inhibitor (fig. S8, A to E). Corroborating the pathway analysis, MYC and E2F ChIP-seq revealed attenuated binding of these TFs at target gene promoters after treatment of Q901 (Fig. 4, B and C, and fig. S9A).

**Fig. 4.**
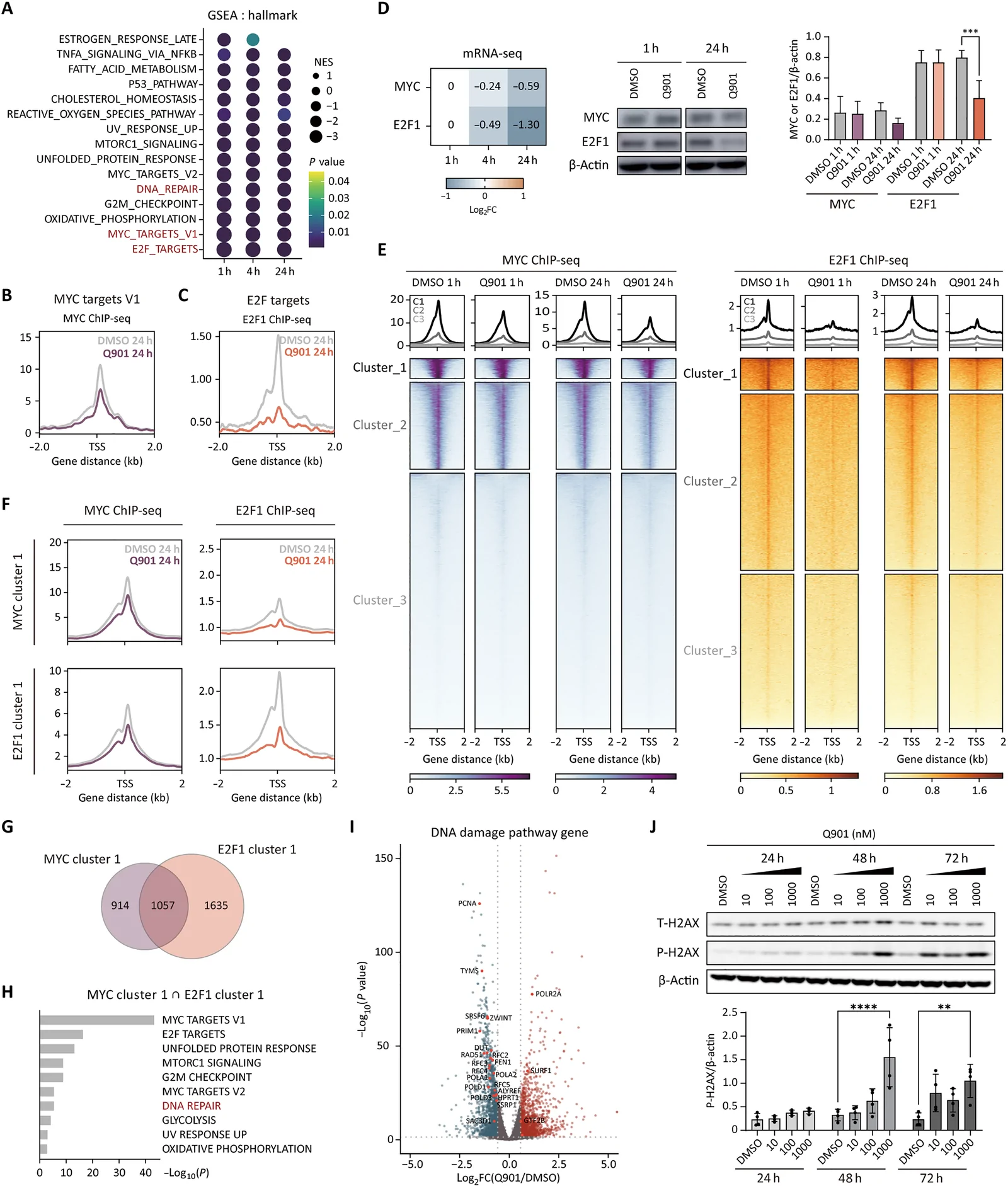
Q901 disrupts MYC-E2F-DDR transcriptional axis. (**A**) GSEA of pan RNAPII ChIP-seq data following Q901 treatment (*n* = 2; fgsea *P* value ≤ 0.05). NES, normalized enrichment score. (**B**) Average MYC ChIP-seq signal plot of the MYC target V1 gene set (*n* = 2). (**C**) Average E2F1 ChIP-seq signal plot of the E2F target V1 gene set (*n* = 2). (**D**) Heatmap showing log_2_FC values of MYC and E2F1 expression from mRNA-seq data and Western blot showing MYC and E2F1 protein levels with or without Q901 treatment (right; *n* = 3; two-way ANOVA followed by Šidák’s multiple comparison, data represent means ± SD; ***P ≤ 0.001). (**E**) Average plots and heatmaps showing MYC ChIP-seq signals for MYC-clustered genes (left) and E2F1 ChIP-seq signals for E2F1-clustered genes (right). (**F**) Average plots of MYC and E2F1 ChIP-seq signals for MYC cluster 1 and E2F1 cluster 1 genes. (**G**) Venn diagram showing the overlap between MYC cluster 1 and E2F1 cluster 1 protein-coding genes. (**H**) GO analysis of protein-coding genes commonly present in MYC cluster 1 and E2F1 cluster 1. (**I**) Volcano plot of mRNA-seq signals after Q901 24-hour treatment (*n* = 3; blue dot indicates *P* value ≤ 0.05 and log_2_FC ≤ −0.58; red dot indicates *P* value ≤ 0.05 and log_2_FC ≥ 0.58). DNA damage pathway genes indicated by a red dot and labeled. (**J**) Western blot data of total H2AX and its phosphorylated form of H2AX (γH2AX) after Q901 treatment for indicated times (*n* = 4; ordinary one-way ANOVA followed by Šidák’s multiple comparison, data represent means ± SD; **P ≤ 0.01, ****P ≤ 0.0001).

To investigate the mechanism by which CDK7 inhibition modulates the binding properties of MYC and E2F, we quantified changes in their expression levels at 1 and 24 hours post–Q901 treatment (Fig. 4D). Only the E2F protein level showed a reduction at the 24-hour time point (Fig. 4D, middle and right). Therefore, the diminished binding of both TFs observed as early as 1 hour following Q901 treatment cannot be solely attributed to changes in protein levels. We also observed no alterations in the phosphorylation status of MYC at residues S62 and T58, which have been previously implicated in MYC protein stability (fig. S9B) (*33*). The retinoblastoma protein (Rb) is known to exert cell cycle suppression through direct interaction with E2F, thereby sequestering it (*34*). Phosphorylation of Rb at these specific residues triggers the dissociation of E2F, facilitating the transcriptional activation of genes essential for cell cycle progression. However, Q901 treatment did not induce notable changes in Rb phosphorylation at residues S780 and S795, suggesting that changes in Rb-mediated E2F sequestration are unlikely to cause the observed reduction in E2F binding to target gene promoters (fig. S9B). We also found no changes in protein levels of MYC dimerization partner, MAX (fig. S9C). Therefore, inhibition of key oncogenic pathways by Q901 appears to be mediated by both altering the TF binding properties and the expression levels during the course of CDK7 inhibition.

### CDK7 inhibition impairs DNA repair pathway by controlling MYC and E2F1 activity

MYC and E2F are key TFs involved in cell cycle progression, cell growth, and proliferation, which are crucial for cancer progression and survival. To gain additional molecular insight into the effect of Q901-mediated CDK7 inhibition in MYC and E2F activity, the entire genes were ranked on the basis of MYC or E2F1 occupancy levels at the TSS regions, and the highest occupancy cluster (cluster 1) for each TF was compared for Q901 effects (Fig. 4E and fig. S9D). Both TFs showed strong binding at cluster 1 and exhibited reduced occupancy upon Q901 treatment (Fig. 4, F and G, and fig. S9E), suggesting extensive cooperation between MYC and E2F1 in MCF-7 cells. They also bound most SEs while occupying only about half of TEs (fig. S10, A to C). Q901 substantially reduced TF occupancy levels at enhancers as well as their target genes (fig. S10, B and C). These results collectively suggests that Q901 could suppress the MYC- and E2F-driven oncogenic transcription program by reducing their binding activities at both promoters and enhancers.

GO analysis of cluster 1 genes enriched for both TFs identified multiple pathways that were also substantially altered upon CDK7 inhibition in the GSEA of the full gene set, suggesting that Q901 primarily affects genes associated with MYC and E2F1 regulatory programs (Fig. 4, A and H). We next focused on DNA damage repair (DDR) genes as DNA damage accumulation is a well-established mechanism underlying anticancer activity (*35*). Most DDR genes were down-regulated following Q901 treatment (Fig. 4I and fig. S11, A and B). Consistent with this, γH2AX levels increased over time in a dose-dependent manner, indicating enhanced DNA damage (Fig. 4J). Therefore, Q901 can lead to DDR deficiency in MCF-7 cells by altering transcription activity of MYC and E2F at both promoters and enhancers and subsequent down-regulation of genes involved in the DDR pathway.

### Q901 stabilizes TOP1-DPCs

TOP1-DPCs represent a unique type of DNA lesion that can arrest RNAPII elongation complexes. Upon collision with a TOP1-DPC, RNAPII undergoes ubiquitination and 26*S* proteasome–mediated degradation, which also facilitates removal of the TOP1-DPC and activates TCR (*17*). Failure to resolve these lesions ultimately leads to cell death (*17*, *18*). A recent study in SCLC cells has shown that THZ1, another CDK7 inhibitor, enhances the anticancer activity of TPT by promoting ubiquitin-mediated degradation of RNAPII, thereby preventing proteasomal removal of TOP1-DPCs (*25*). Consistent with this mechanism, and on the basis of our observation that Q901 reduces promoter-proximal RNAPII (Fig. 2C), we hypothesized that Q901 similarly enhances TPT activity by limiting the pool of elongating RNAPII available to engage TOP1-DPCs and trigger their removal.

To directly test whether Q901 prolongs TOP1-DPC persistence, we performed TOP1cc-seq (TOP1 cleavage complex sequencing) and γH2AX ChIP-seq following drug washout. MCF-7 cells were treated with TPT alone or TPT+Q901 for 30 min, followed by drug washout and sample collection at 0, 15, and 30 min. TOP1cc-seq was conducted without formaldehyde cross-linking using an antibody specific for TOP1-DNA covalent complexes, enabling selective detection of enzymatically trapped TOP1-DPCs rather than noncovalently chromatin-associated TOP1. Because active transcription generates torsional stress that promotes TOP1 engagement, we compared active and inactive genes classified on the basis of RNAPII occupancy. Active genes exhibited substantially higher TOP1-DPC signals than inactive genes (Fig. 5A). Under the combination treatment (Q901+TPT), TOP1-DPC signals progressively increased following washout, whereas the TPT-only condition showed a modest decline over time. Consistent with these findings, TOP1 protein levels progressively decreased following TPT treatment, whereas this reduction was attenuated by Q901 cotreatment (fig. S12, A and B). This supports a model in which collision with elongating RNAPII promotes TOP1 degradation under TPT alone, whereas CDK7 inhibition reduces such collisions, thereby stabilizing TOP1-DPCs and contributing to their accumulation over time.

**Fig. 5.**
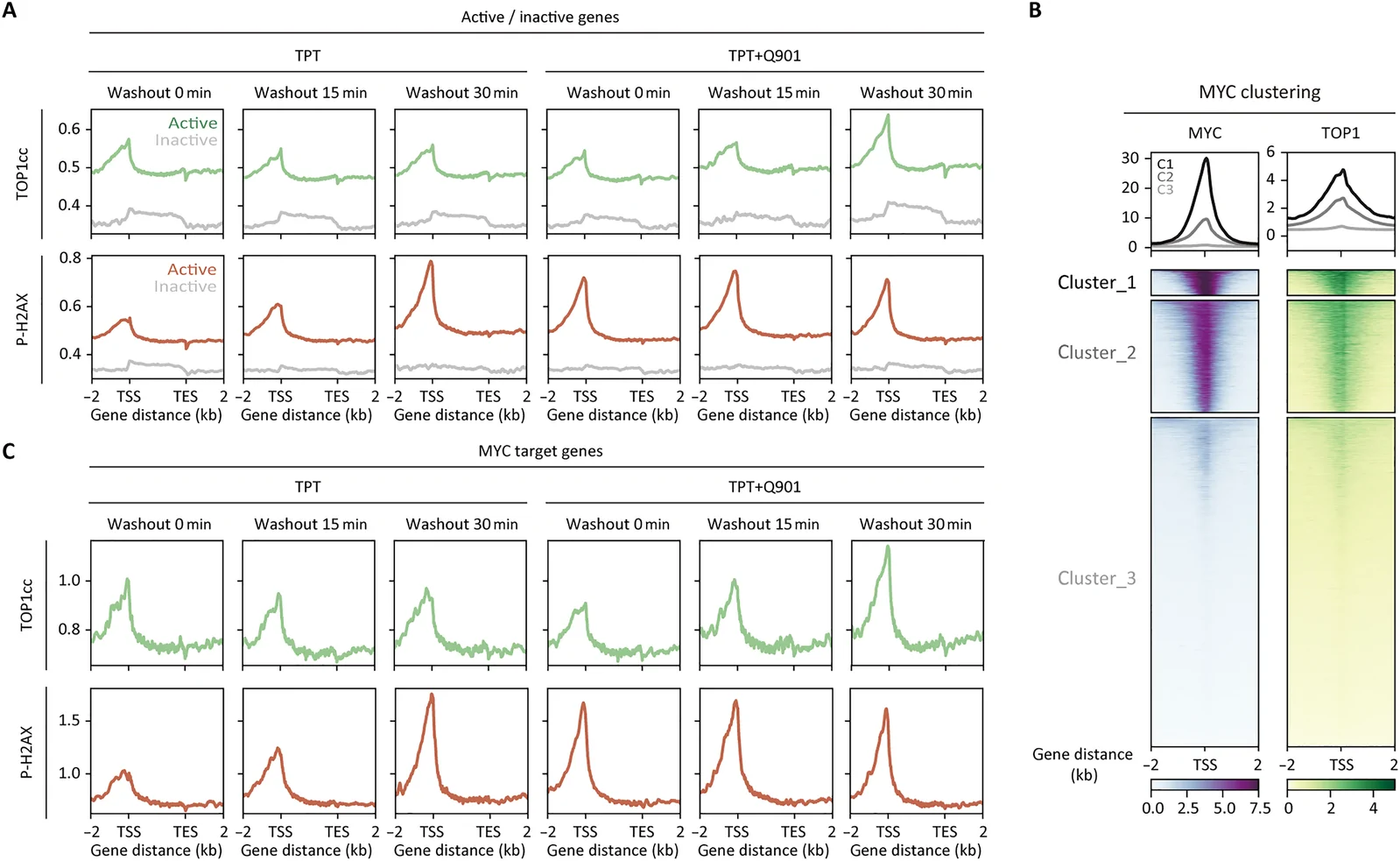
Q901 reduces the TSS-associated TOP1-MYC complex and prolongs TOP1-DPC stability. (**A**) Average TOP1cc-seq and γH2AX ChIP-seq signal plot of active genes (pan RNAPII signal > 1) and inactive genes (*n* = 2). (**B**) Average plots and heatmaps of MYC and TOP1 ChIP-seq signals at three gene clusters ranked by MYC binding levels (DMSO condition). (**C**) Average TOP1cc-seq and γH2AX ChIP-seq signal plot of MYC target genes (*n* = 2).

MYC is known to alleviate transcription-associated torsional stress by recruiting TOP1 and topoisomerase II (TOP2) at promoters, gene bodies, and enhancers (*36*). Accordingly, TOP1 occupancy closely tracked MYC binding across target genes (Fig. 5B and fig. S12, C and D), and TOP1cc-seq profiles showed similar enrichment patterns at MYC targets and transcriptionally active genes (Fig. 5, A and C). Together, these findings indicate that MYC-driven transcriptional activity marks sites of elevated TOP1 engagement and TOP1-DPC formation and further support a model in which CDK7 inhibition prolongs TOP1-DPC persistence at these highly transcribed regions.

RNA sequencing (RNA-seq) analysis further revealed that Q901 and TPT cooperatively suppress DDR pathways. A set of 17 DDR genes, including homologous recombination (RAD51, BRCA2, XRCC5, and XRCC6), checkpoint (CHEK1, CHEK2, and WEE1), and replication/repair factors (PCNA, LIG1, PRIM1, POLA1, and POLA2), was substantially down-regulated under combination treatment (*P* ≤ 0.05) (fig. S11C).

Collectively, these results support a unified model in which CDK7 inhibition constrains RNAPII elongation, thereby reducing RNAPII–TOP1-DPC collisions that would otherwise trigger proteasomal clearance. This leads to the persistence of TOP1-DPCs, which, together with suppression of DDR pathways, amplifies DNA damage and enhances cytotoxicity.

### A combination of Q901 and TOP1 inhibitors enhances antitumor efficacy

Our study demonstrates that Q901-mediated CDK7 inhibition enhances the efficacy of TOP1 inhibitors through three distinct mechanisms: (i) reducing MYC and E2F recruitment to oncogenic transcription programs, (ii) impairing DDR pathways by down-regulating DDR genes, and (iii) prolonging TOP1-DPCs by blocking their proteasomal degradation through RNAPII displacement (Fig. 6A).

**Fig. 6.**
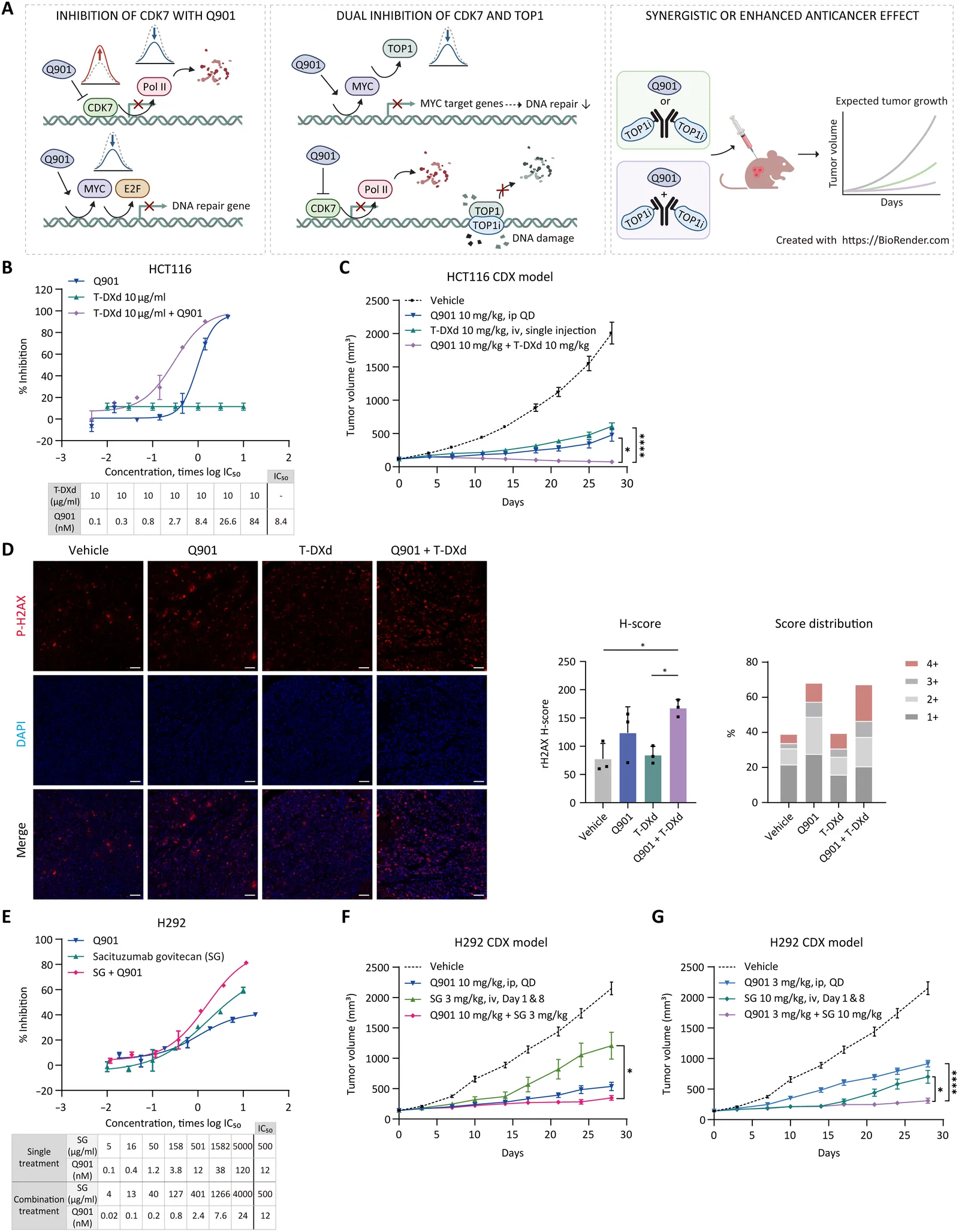
Combining Q901 with TOP1i-ADCs enhances antitumor effects. (**A**) Model illustrating how Q901 enhances the activity of TOP1i and TOP1i-ADCs. (Left) Q901 promotes CDK7 accumulation at the TSS while reducing RNAPII, MYC, and E2F1 binding, leading to down-regulation of DNA damage response genes. (Middle) The dual inhibition of CDK7 (by Q901) and TOP1 (by TOP1i) blocks the repair of TOP1i-induced DNA damage, leading to cell death. (Right) The combination of Q901 and a TOP1i-ADC shows in vitro cytotoxicity and in vivo tumor suppression. (**B** and **C**) HCT116 cells were treated with Q901, T-DXd (10 μg/ml), or the combination for 72 hours (B). Dose-response curves were generated from log-transformed concentration relative to IC_5__0_. Cell viability was measured using the ATP Lite system (*n* = 2, data represent means ± SD). (C) HCT116 xenograft models were treated with Q901 (10 mg/kg), T-DXd (10 mg/kg), or the combination (*P ≤ 0.05, **P ≤ 0.0001 by a two-way ANOVA followed by Tukey’s multiple comparisons test). CDX, cell line–derived xenograft; ip, intraperitoneally; iv, intravenously. (**D**) In vivo γH2AX IF analysis showing representative images [left; γH2AX, 4′,6-diamidino-2-phenylindole (DAPI), and merged; scale bars, 20 μm], H-score (middle; two-way ANOVA followed by Šidák’s multiple comparison, data represent means ± SD; *P ≤ 0.05), and score distribution (right). (**E** to **G**) H292 cells were treated with Q901 in combination with SG for 72 hours (E). Dose-response curves were generated from IC_5__0_-normalized log-transformed concentrations. Cell viability was assessed using the ATP Lite system (*n* = 2, data represent means ± SD). [(F) and (G)] H292 xenograft models were treated with Q901 (10 or 3 mg/kg), SG (3 or 10 mg/kg), or the combination. The tumor volume is presented as means ± SEM (*P ≤ 0.05, ****P ≤ 0.0001 by a two-way ANOVA followed by Tukey’s multiple comparison test).

To validate the proposed mechanisms across multiple cancer models, we first examined rapidly proliferating neuroendocrine SCLC cell lines, H82 and DMS53. SCLC was chosen as a clinically relevant model to evaluate the drug combination, particularly given that TOP1 inhibitors such as TPT, are already used in clinical settings for SCLC treatment. As expected, the combined administration of Q901 and TPT for 72 hours resulted in enhanced antiproliferative activity compared to either drug alone (fig. S13, A and B).

To further examine whether Q901 can enhance TOP1 inhibitor efficacy in a less sensitive context, we tested H446 cells, an SCLC-N subtype cell line that exhibits intrinsically reduced sensitivity to TPT compared to the SCLC-P subtype H526 cells. Previous pharmacologic profiling studies have demonstrated that SCLC-N subtypes are generally less responsive to DNA damage–targeting agents (*37*). The combination of Q901 and TPT showed a favorable combination effect in H446 cells, as indicated by combination index (CI) analysis (CI < 1; fig. S13C), suggesting that CDK7 inhibition can enhance TOP1 inhibitor efficacy even in cells with reduced intrinsic sensitivity. Furthermore, to examine whether the combination effect depends on covalent CDK7 inhibition, we performed cell viability assays combining TPT with either Q901 or the noncovalent analogs Q901R and QS0551508. Consistent with the ChIP-seq results (fig. S5F), a clear combination effect with TPT was observed only with Q901, whereas the noncovalent analogs did not exhibit comparable responses (fig. S13D). These findings indicate that CDK7 inhibition with covalent engagement, rather than reversible catalytic inhibition alone, is essential for the enhanced sensitivity to TOP1 inhibitors.

We next examined whether Q901 could enhance the efficacy of T-DXd, a HER2-targeting ADC carrying a TOP1 inhibitor payload. Because cellular uptake of the TOP1 inhibitor component of T-DXd depends on HER2 abundance (*20*, *22*, *38*), we focused on a system with low HER2 expression to assess the combination’s efficacy under unfavorable delivery conditions. We selected the HCT116 colon cancer model, which exhibits ultralow HER2 expression and shows no response to T-DXd in cellular cytotoxicity assays, likely due to insufficient intracellular delivery of DXd (Fig. 6B). HCT116 cells were insensitive to T-DXd monotherapy (10 μg/ml), whereas Q901 alone inhibited cell growth with an IC_50_ of 8.4 nM. We then evaluated drug interactions using the combination analysis workflow described by Uitdehaag *et al.* (*38*), which assesses efficacy across multiple drug ratios. Notably, combining a fixed, inactive dose of T-DXd (10 μg/ml) with increasing concentrations of Q901 substantially enhanced growth inhibition across most ratios tested. These results indicate that Q901 sensitizes cells to DXd at doses that are otherwise noncytotoxic.

Next, the in vivo antitumor activity of this combination was evaluated in HCT116 xenograft models. Mice bearing HCT116 tumors were treated with T-DXd (10 mg/kg), Q901 (10 mg/kg), or their combination (Fig. 6C). By day 28, T-DXd and Q901 monotherapies achieved similar tumor growth inhibition (TGI) of 74 and 81%, respectively. Notably, despite the lack of response to T-DXd in cellular assays at 72 hours, substantial TGI was observed in vivo. This discrepancy can be attributed, in part, to systemic circulation release of DXd payload from circulating T-DXd, leading to free payload-mediated antitumor effects (*39*). To test this possibility, T-DXd was incubated in the mouse serum for 21 days, and the drug-to-antibody ratio (DAR) was measured. The results showed a marked decrease in DAR values by day 21, indicating that extensive DXd payload release into the mouse serum is a likely cause of the discrepancy between the cellular assay and the mouse xenograft test (fig. S14A). Although systemic payload release may contribute to the observed in vivo activity, extended exposure of T-DXd over 21 days in vivo may also contribute to the activity. Notably, extending T-DXd treatment to 120 hours in the cellular assay reduced cell viability, whereas no such effect was observed at 72 hours (fig. S14B). Nevertheless, the combination treatment enhanced the efficacy of tumor suppression with 102% TGI. Although tumors in the monotherapy groups resumed growth ∼11 days posttreatment and gradually increased in size, the Q901/T-DXd combination exhibited tumor suppression throughout the study duration.

As HCT116 xenograft models showed enhanced TGI with combination treatment, this model was used to assess whether DDR suppression translates into increased DNA damage in vivo. γH2AX immunofluorescence (IF) was performed on tumors treated with Q901, T-DXd, or their combination (Fig. 6D). Combination treatment substantially increased γH2AX H-score compared to T-DXd alone (Fig. 6D, left and middle) and elevated the proportion of cells with high foci counts (H-score of 4+), indicating enhanced DNA damage accumulation (Fig. 6D, right).

Together with the in vitro and in vivo data, these results support a model in which Q901 enhances DNA damage accumulation by prolonging TOP1-DPC persistence and suppressing DDR pathways, thereby contributing to the improved therapeutic efficacy observed in preclinical models.

To further extend our findings, we investigated another TOP1i-ADC, sacituzumab govitecan (SG), which targets trophoblast cell surface antigen 2 (TROP2)—a transmembrane glycoprotein frequently overexpressed in many epithelial malignancies and associated with aggressive tumor behavior. SG binds to TROP2 on the tumor cell surface, undergoes receptor-mediated internalization, and is cleaved intracellularly to release SN-38, a potent TOP1 inhibitor that induces DNA damage in tumor cells (*40*, *41*). The TROP2-positive cell line was used to evaluate the combination effect of Q901 under conditions where SG is expected to be active. In TROP2-positive H292 lung cancer cells, both SG (IC_50_ of 500 ng/ml) and Q901 (IC_50_ of 12 nM) as monotherapies inhibited cell proliferation, but their combination substantially increased the antiproliferative effect (Fig. 6E). In H292 xenograft models, two dosing regimens were evaluated. In the first, SG monotherapy at 3 mg/kg achieved 47% TGI and Q901 monotherapy at 10 mg/kg achieved 80% TGI by day 28. The combination treatment enhanced the efficacy to 90% TGI (Fig. 6F). In the second regimen, SG monotherapy at 10 mg/kg achieved 72% TGI and Q901 monotherapy at 3 mg/kg achieved 61% TGI by day 28. The combination treatment enhanced efficacy to 92% TGI (Fig. 6G). Notably, tumors treated with SG alone showed regrowth after 14 days, whereas both Q901 monotherapy and the combination treatment maintained sustained TGI throughout the study period, with the combination showing slightly higher efficacy. Collectively, these results highlight Q901 as a promising combination partner for TOP1 inhibitors and TOP1i-ADCs across multiple cancer models. Q901 not only enhances efficacy in tumors already responsive to TOP1i-ADCs but also provides benefit in setting with limited ADC activity, such as those with low antigen expression or reduced TOP1 inhibitor exposure due to suboptimal dosing.

## DISCUSSION

Our study presents Q901 as a highly selective CDK7 inhibitor with potential to address challenges in cancer therapy, particularly in combination with TOP1 inhibitors. By targeting CDK7, Q901 traps CDK7 at promoter-proximal regions, preventing RNAPII from progressing into elongation. In MCF-7 cells, CDK7 inhibition primarily disrupts oncogenic transcription programs driven by MYC and E2F and impairs the DNA damage response pathway. These effects enhance tumor sensitivity to TOP1 inhibitors by prolonging DNA damage caused by TOP1-DPCs. These findings underscore the therapeutic promise of Q901, especially in cancers with high transcriptional activity and limited response to current TOP1 inhibitor–based therapies.

Preclinical studies of CDK7 inhibitors have demonstrated anticancer activities through cell cycle arrest and suppression of transcription (*12*–*14*). Although cell cycle arrest was commonly observed with all tested inhibitors, their effects on transcription and mechanisms of action varied depending on the type of inhibitor used. THZ1 and its derivatives (SY-1365 and SY-5609) induce G_2_-M cell cycle arrest and apoptosis, exhibiting strong anticancer effects against various cancer types (*13*). Global transcription repression was also observed, accompanied by a loss of RNAPII CTD phosphorylation (*12*, *13*, *15*). The extreme sensitivity of T cell leukemia (T-ALL) cells to THZ1 was attributed to transcription addiction, especially those genes controlled by SEs and often driven by oncogenic TFs like MYC (*13*). However, the observed anticancer effects through transcriptional inhibition were later found to be contributed by off-target inhibition of CDK12/13, rather than through CDK7 inhibition alone (*12*, *13*, *15*).

In contrast, YKL-5-124, a highly selective CDK7 inhibitor with no measurable affinity for CDK12/13, showed transcriptional repression in a relatively limited set of gene expression programs, including E2F and MYC pathways, without broadly affecting SE-mediated or global transcription. Treatment with YKL-5-124 did not result in reductions in bulk RNAPII phosphorylation and primarily induced G_1_ phase cell cycle arrest without noticeable evidence of apoptosis (*14*). These findings collectively suggested a nonessential role for CDK7 in regulating RNAPII CTD phosphorylation and global basal gene expression. However, it causes DNA replication stress and genomic instability, which, in turn, activate antitumor immune responses by promoting lymphocyte activation (*42*).

Q901 exhibits high selectivity for CDK7 over other CDKs (Fig. 1). Its anticancer effects are being confirmed in the preclinical as well as in clinical studies. Exhibiting no off-target effects in CDK12/13 (Fig. 1), the selectivity of Q901 appears to be similar to that of YKL-5-124, yet our study delineates more defined transcriptional mechanisms elicited by specific CDK7 inhibition, some of which had not been previously appreciated. Q901 substantially decreased the levels of chromatin-bound RNAPII and CTD phosphorylation at thousands of genes (Fig. 2, B and C). Notably, catalytically inhibited CDK7 became trapped at the promoter-proximal regions (Fig. 2D). These findings are in line with a recent study demonstrating the direct role of CDK7 in RNAPII promoter escape by facilitating initiation factor release (*28*). Rapid CDK7 inhibition caused RNAPII retention at promoters, leading to decreased RNAPII initiation and global down-regulation of transcription. We envision that trapping of catalytically inhibited CDK7 appears to be the key mechanism for the retention of RNAPII initiation complex at the promoter-proximal regions. Q901 also altered the enhancer activity by reducing RNAPII binding and eRNA transcription at both SEs and TEs, leading to impaired transcription from their predicted target genes. In contrast with the preferential inhibitory activity of THZ1 on oncogenic programs driven by SEs, there was no bias in the effect of Q901-mediated CDK7 inhibition toward altering SE activities compared to TEs in MCF-7 cells (Fig. 3). Nonetheless, enhancer target genes affected by Q901 were enriched in pathways related to nucleosome organization and translation, suggesting that the anticancer effect of Q901 is, in part, mediated by suppressing enhancer activities (fig. S6E).

Our systematic analysis identified that MYC and E2F occupied the promoters of nearly all expressed genes in MCF-7 cells, with their occupancy levels being highly correlated with transcription levels (Fig. 4E). Q901 globally decreased their binding levels (Fig. 4, E and F). MYC can promote transcription through multiple mechanisms such as facilitation of the RNAPII pause-release and the recruitment and activation of TOP1 to alleviate DNA topological stress during active transcription (*36*). Therefore, attenuated MYC binding would further contribute to the anticancer activity of Q901.

ADCs represent a rapidly expanding class of anticancer therapeutics, with 14 FDA-approved agents on the market. TOP1 inhibitors are increasingly used as payloads to enhance both the therapeutic index and targeted delivery to antigen-expressing tumors as they generally exhibit manageable toxicity compared to other anticancer agents while remaining highly effective in cancers that require high levels of transcription and replication (*19*). Besides already approved T-DXd (Enhertu) and SG, more than 100 TOP1i-ADC candidates are currently in clinical trials (*40*). Despite their promising potential, the development of new TOP1i-ADCs faces challenges, primarily due to two factors: (i) the need for an increased therapeutic window and (ii) the necessity of overcoming cross-resistance issues when treating patients with ADCs in sequence. Toxicities of TOP1i-ADCs often resemble those of the standalone TOP1 inhibitor chemotherapies. To improve the toxicity profile of ADCs, multiple strategies are being used, especially through advancements in ADC engineering such as site-specific conjugation, the use of stable linkers, and hydrophobicity masking technologies (*41*). However, these engineering approaches have yet to demonstrate clinical benefits (*40*). With numerous ADCs under clinical development, there is a concern of cross-resistance when a patient with cancer is treated with sequential use of ADCs (*40*). Although limited, some clinical data suggest that activation of DDR may be a common mechanism driving resistance to sequential TOP1i-ADC treatments (*43*, *44*). Our study identified Q901 as a promising combination partner for TOP1 inhibitors and TOP1i-ADCs across multiple preclinical cancer models (Fig. 6A). Mechanistically, Q901-mediated CDK7 inhibition suppressed DDR pathways by transcriptionally regulating MYC and E2F activity (Fig. 4). In addition, it prevented the proteasomal degradation of TOP1-DPCs by disrupting the transition of RNAPII complex into elongation (Fig. 5).

Our study strongly supports the combinatorial use of Q901 and TOP1i-ADCs as a novel strategy to enhance ADC efficacy and broaden their therapeutic potential in cancer treatment. This approach may be particularly beneficial in settings where ADC activity is limited, such as in tumors with low antigen expression that restricts TOP1 inhibitor exposure, when dose reductions are required to manage toxicity, or when multiple TOP1i-ADCs are administered sequentially.

## MATERIALS AND METHODS

### Cell lines and reagents

MCF-7, H82, HCT116, H292, and H446 cell lines were purchased from the American Type Culture Collection (ATCC) (Manassas, VA, USA). A2780 was purchased from the ECACC (Porton Down, Wiltshire, UK). Cells were confirmed to be pathogen-free (Mycoplasma testing, Lonza, L108-318). MCF-7 cells were maintained in Dulbecco’s modified Eagle’s medium (DMEM) (Thermo Fisher Scientific, Waltham, MA, USA, Gibco, 31053028) with 10% fetal bovine serum (FBS) (Invitrogen, Carlsbad, CA, USA, 10091148), GlutaMAX (Thermo Fisher Scientific, Gibco, 35050061), and penicillin-streptomycin (Welgene, Gyeongsansi, South Korea, LS202-02), and for short-term treatment, DMEM (Gibco, 31053028) with 10% charcoal-stripped FBS (Gibco, A3382101) and GlutaMAX (Gibco, 35050061) was used. HCT116 were maintained in McCoy’s 5A medium (ATCC, 30-2007) with 10% FBS (Invitrogen, 10091148), DMS53 were maintained in Waymouth’s MB 752/1 medium (Merck, Darmstadt, Germany, W1625) with 10% FBS (Invitrogen, 10091148), and H82, H292, A2780, and H446 were maintained in RPMI 1640 (ATCC, 30-2001) with 10% FBS (Invitrogen, 10091148). Cells were cultured at 37°C and 5% CO_2_. All animal experiments were performed in compliance with protocols approved by the Institutional Animal Care and Use Committee [IACUC approval no. ON01-SH006-2023v1.1; animal facility license no. SYXK (沪) 2020-0034] of WuXi AppTec and complied with the guidelines by the Association for Assessment and Accreditation of Laboratory Animal Care (AAALAC). Q901, QS0551546 (Q901R), QS0551508, and Bio-QS (Bio-QS1169; a biotinylated Q901 analog) were synthesized by Qurient (Seongnam-si, South Korea). TPT was purchased from Sigma-Aldrich (St. Louis, MO, USA, T2705). T-DXd and SG were purchased from Evidentic GmbH (Potsdam, Germany).

The following antibodies were used for immunoblotting: total CDK7 (Cell Signaling Technology, Danvers, MA, USA, 2090S, RRID: AB_2077140), MYC (Cell Signaling Technology, 13987, RRID: AB_2631168), E2F1 (Cell Signaling Technology, 3742, RRID: AB_2096936), MYC S62P (Cell Signaling Technology, 13748, RRID: AB_2687518), MYC T58P (Cell Signaling Technology, 46650), Rb S795P (Cell Signaling Technology, 9301, RRID: AB_330013), Rb S780P (Cell Signaling Technology, 9307, RRID: AB_330015), MAX (Cell Signaling Technology, 4739, RRID: AB_2281777), T-H2AX (Santa Cruz, Dallas, TX, USA, sc-517336, RRID: AB_3675923), γH2AX (Cell Signaling Technology, 2577, RRID: AB_2118010), CHK1 (Cell Signaling Technology, 2360S, RRID: AB_2080320), p-CHK1 (Ser^345^) (Cell Signaling Technology, 2348S), RAD51 (Cell Signaling Technology, 8875S), EXO1 (Cell Signaling Technology, 63862S), FANCD2 (Cell Signaling Technology, 16323S), and RFC4 (Abcam, Cambridge, UK, ab182145), β-actin (Santa Cruz, sc-47778, RRID: AB_626632), Affinity Purified Goat Anti-Rabbit IgG (H+L)-HRP (Bio-Rad, Hercules, CA, USA, 1706515, RRID: AB_11125142), and Affinity Purified Goat Anti-Mouse IgG (H+L)-HRP (Bio-Rad, 1706516, RRID: AB_11125547).

The following antibodies were used for ChIP-seq: pan RNAPII (Cell Signaling Technology, 2629 RRID: AB_2167468), Ser5P (Abcam, ab5408, RRID:AB_304868), Ser2P (Abcam, ab5095, RRID:AB_304749), CDK7 (Bethyl, Montgomery, TX, USA, A300-405A, RRID: AB_2275973), MYC (Cell Signaling Technology, 13987, RRID: AB_2631168), E2F1 (Cell Signaling Technology, 3742, RRID: AB_2096936), Anti-Topoisomerase I-DNA Covalent Complexes (clone 1.1A) (Sigma-Aldrich, MABE1084, RRID: AB_2756354), and Anti-phospho-H2A.X (Ser^139^) (Merck Millipore, Burlington, MA, USA, 07-164, RRID: AB_11213838).

### Inhibitor treatment

To assess the durability of CDK7 occupancy after Q901 removal, cells were treated with 6 nM Q901 for 4 hours, washed with fresh medium, and incubated for 4, 24, or 48 hours. For the short-term condition, MCF-7 cells were treated with DMSO or 100 nM Q901 for 1 hour followed by 1 hour of ethanol (EtOH)/E2 (estrogen) treatment (the E2 condition was not used in this study). For long-term treatment, MCF-7 cells were treated with DMSO or 100 nM Q901 for 4, 24, 48, or 72 hours. For the detection of TOP1 protein from whole-cell lysates, MCF-7 cells were pretreated with DMSO or Q901 100 nM for 2 hours, followed by treatment with TPT 1 μM for 0, 1, 6, or 24 hours. For TOP1 and MYC ChIP-seq, MCF-7 cells were pretreated with Q901 100 nM for 2 hours, followed by treatment with TPT 1 μM for 6 hours. For TOP1cc-seq and γH2AX ChIP-seq, MCF-7 cells were treated with 1 μM TPT alone or 1 μM TPT combined with 100 nM Q901 for 30 min, followed by washout and sample collection at 0, 15, and 30 min.

### NMR analysis

^1^H nuclear magnetic resonance (NMR) spectrum was recorded at 298 K (VT-NMR at 353 K) using a Bruker Avance III HD 400 MHz NMR spectrometer equipped with a broadband observe (BBO) probe (Bruker BioSpin GmbH, Rheinstetten, Germany). Chemical shifts (δ) are reported in parts per million (ppm), calibrated to residual nondeuterated solvent signals. Multiplicities are abbreviated as s (singlet), d (doublet), t (triplet), q (quartet), m (multiplet), and br (broad). Coupling constants (*J*) are reported in hertz (Hz), and integrals are provided for ^1^H NMR spectra.

### Inhibition profile of 410 kinases and titration of 28 CDKs

A radiometric protein kinase assay service was provided by Reaction Biology (Freiburg, Germany) to measure the kinase activity of 410 protein kinases and 28 CDKs.

In the kinase inhibition assay, the residual activity for each compound was calculated using the following formula: Residual activity (%) = 100 × [(signal of compound − low control)/(high control − low control)]. The selectivity score of the compound at the tested concentrations was calculated for residual activity < 50%, that is, inhibition of >50%. The selectivity score for a particular compound at a particular concentration was calculated using the following formula: Selectivity score = (count of data points < 50%)/(total number of data points).

In the 28 CDK titration assay, the residual activity and IC_50_ values of the compound were calculated using the Quattro Workflow V3.1.1 (Quattro Research GmbH, Munich, Germany). The ATP concentration used in each kinase reaction was set to the apparent ATP-*K*_m_ value. Dose-response curve fitting was performed using a four-parameter logistic regression model in GraphPad Prism 10.4.1 (GraphPad Software, Boston, MA, USA).

### Q901 covalent binding site evaluation

The recombinant CAK complex (CDK7/cyclin H/MAT1; Thermo Fisher Scientific, PV3868) was incubated with Q901 or DMSO for 1 hour at room temperature (RT), and the reaction complexes were resolved by SDS–polyacrylamide gel electrophoresis (PAGE). The bands corresponding to CDK7 were excised for in-gel digestion. Denaturation, reduction, and alkylation were performed using 8 M urea, TCEP, and iodoacetamide at RT, followed by Arg-C digestion (1:100 Arg-C:protein ratio, at 37°C for 18 hours) and Arg-C/trypsin digestion (1:100 Arg-C:protein ratio and 1:50 trypsin:protein ratio, at 37°C for 18 hours). The extracted peptides were loaded and eluted using a high-performance liquid chromatography (HPLC) system and then separated through a linear gradient (3 to 60% mobile phase B in 120 min) at a flow rate of 0.3 μl/min using EASY-Spray HPLC Columns (75 μm/500 mm) (Thermo Fisher Scientific, ES903) at 40°C and introduced into an Orbitrap Exploris 240 LC-MS/MS System (Thermo Fisher Scientific, BRE725535) in positive ion mode and run in data-independent acquisition (DIA) mode. Data acquisition was performed over a mass/charge ratio (*m/z*) range of 390 to 1010, and peak integrations for fragment ions of the most intense charge state of the doubly carbamidomethylated Cys^312^-containing peptide were analyzed using the Skyline software 24.1 (Seattle, WA, USA). The carbamidomethylated Cys^304^-containing CDK7 peptide, PGPTPGCQLPR (residues 299 to 309), was used for normalization.

### Enzymatic assay

CDK7 kinase activity was measured using a LANCE Ultra TR-FRET kinase assay (Revvity, formerly PerkinElmer, Waltham, MA, USA) according to the manufacturer’s protocol. Briefly, recombinant human CDK7/cyclin H/MNAT1 His-tag protein (Invitrogen, PR6749B) was incubated with compounds (Q901, Q901R, and QS0551508) in kinase buffer in a 384-well format. The assay used a ULight-labeled peptide substrate and a europium-labeled anti-phospho antibody for time-resolved fluorescence resonance energy transfer (TR-FRET) detection.

TR-FRET signals were measured using an EnVision multimode plate reader (Revvity, EnVision 2105 Alpha) with LANCE/DELFIA detection settings.

Compounds were tested using an eight-point serial dilution (top concentration: 3000 nM, 1:4 dilution). Signals were normalized to DMSO-treated controls to calculate residual kinase activity (%), and dose-response curves were generated using GraphPad Prism 10.4.1.

### CellTiter-Glo assay

Cells were seeded into 96-well plates and treated with compounds at the indicated concentrations and time points. After incubation, cell viability was measured using either CellTiter-Glo luminescent cell viability assay (Promega, Madison, WI, USA, G7572) according to the manufacturer’s instructions. Plates were equilibrated to RT for 30 min before adding 50 μl of CellTiter-Glo reagent to each well. Luminescence was measured using the EnVision Multi Label Reader (PerkinElmer, Waltham, MA, USA). Dose-response curves were fitted using a nonlinear regression model with a sigmoidal dose-response function in GraphPad Prism 10.4.1. The absolute IC_5__0_ values were calculated using the dose-response curves generated in XL-fit 5 (IDBS Software, Woking, UK) and fitted with the four-parameter logistic equation: fit = *A* + ((*B* − *A*)/{1 + [(*C*/*x*)*^D^*]}), where *A* and *B* represent the bottom and top plateaus of the curve, respectively, *C* is the IC_5__0_ value, *x* is the compound concentration, and *D* is the Hill slope.

The CI was determined using the Chou-Talalay method (Chou, *Pharmacol. Rev.*, 2006) with the CalcuSyn software (Biosoft, Cambridge, UK). CI values were calculated on the basis of the median-effect equation, where CI < 1 indicates synergism, CI = 1 indicates an additive effect, and CI > 1 indicates antagonism. Normalized isobolograms were generated to visualize the degree of synergism or antagonism at each combination data point.

### CDK7 target occupancy assay

A2780 cells were seeded into 10-cm dishes and treated with Q901 or DMSO as the indicated concentrations for 4 hours. Cells were washed twice with cold phosphate-buffered saline (PBS), lysed with 200 μl of radioimmunoprecipitation assay (RIPA) buffer (Sigma-Aldrich, R0278), and incubated on ice for 30 min. Lysates were scraped and centrifuged at 12,000 rpm for 10 min at 4°C, and protein concentration was determined using a BCA assay. A2780 cell lysates were incubated with 1 μM Bio-QS overnight at 4°C with gentle rocking and then incubated for 3 hours at RT to enhance covalent binding. Bio-QS binds strongly to CDK7 and has the same covalent cassette as Q901 does. After incubation, 200 μl of 50% streptavidin agarose bead slurry (Thermo Fisher Scientific, 20353) was added to the cell lysates, and the samples were incubated overnight at 4°C with gentle rocking for immunoprecipitation (IP). The following day, the samples were pelleted by microcentrifugation (14,000*g*, 30 s, 4°C) and washed three times with ice-cold RIPA buffer, and the supernatant was collected before washing. The pellet was resuspended in 2X SDS sample buffer, vortexed, microcentrifuged for 30 s, heated at 95° to 100°C for 5 min, and centrifuged again at 14,000*g* for 1 min. IP reaction samples were loaded on 4 to 12% Bis-Tris gels as follows: equal volume of IP-reacted samples, input protein, and Bio-QS–incubated lysate for total CDK7 detection. Proteins were transferred to polyvinylidene difluoride (PVDF) membranes, blocked with 5% bovine serum albumin (BSA), and immunoblotted with a CDK7 primary antibody (Cell Signaling Technology, 2090S) followed by a secondary horseradish peroxidase (HRP)–conjugated antibody. Signals were detected using ECL and analyzed using the Image Studio Lite Ver5.2 (LI-COR Biotechnology, Lincoln, NE, USA). Quantitative data of immunoblotting bands were generated by GraphPad Prism 10.4.1.

### Immunoblotting assay

Cell lysates were obtained using RIPA buffer (BIOSESANG, Yongin-si, South Korea, R2002) containing protease inhibitor (GenDEPOT, Baker, TX, USA, P3100) and phosphatase inhibitor cocktails (GenDEPOT, P3200). Samples were collected and kept on ice for 30 min. After centrifugation at 12,000 rpm for 10 min at 4°C, the supernatant was collected for further analysis. Protein concentrations were determined using a Pierce BCA Protein Assay Kit–Reducing Agent Compatible (Thermo Fisher Scientific, Pierce, 23250). Equal amounts of protein were loaded onto SDS-PAGE gels and electrophoresed at 100 V for 1 hour 45 min. Proteins were transferred onto PVDF membranes (MilliporeSigma, Burlington, MA, USA) and blocked with 5% BSA in TBST for 1 hour at RT. Membranes were incubated with primary antibodies overnight at 4°C, washed three times for 10 min each on a shaker, and incubated with HRP-conjugated secondary antibodies for 45 min at RT. The membranes were washed again and soaked in substrate working solution (Merck, WBKLS0100) for 10 s. Chemiluminescent signals were detected using the Image Quant LAS 4000 (PerkinElmer, Waltham, MA, USA). Quantitative data of immunoblotting bands were generated with ImageJ (1.54) and GraphPad Prism 10.4.1.

### Chromatin immunoprecipitation sequencing

ChIP-seq was performed as previously described with minor changes (*45*). After Q901 or TPT was treated, MCF-7 cells were cross-linked for 10 min (MYC, E2F1, TOP1, and γH2AX) or 15 min (other proteins) or left uncross-linked (for TOP1cc-seq) at RT by cross-linking buffer [50 mM Hepes (pH 8.0), 100 mM NaCl, 1 mM EDTA (pH 8.0), and 0.5 mM EGTA (pH 8.0)] with 1% formaldehyde (Sigma-Aldrich, F8775-4X25ML) followed by quenching with 125 mM glycine (Daejung, Siheung-si, South Korea, 4068-4105) for 5 min. Cells were washed with ice-cold 1X PBS, and pellets were stored at −80°C. Frozen pellets were lysed with buffer I [50 mM Hepes (pH 7.5), 140 mM NaCl, 1 mM EDTA (pH 8.0), 10% glycerol, 0.5% NP-40, 1X protease inhibitor (GenDEPOT, P3100), and 1X phosphatase inhibitor (GenDEPOT, P3200)]. The pellets were resuspended in modified buffer II/III [10 mM tris-HCl (pH 8.0), 300 mM NaCl, 0.1% DOC (sodium deoxycholate), 1% Triton X-100, 1 mM EDTA (pH 8.0), 1 mM EGTA (pH 8.0), 1X protease inhibitor, and 1X phosphatase inhibitor] and sonicated at 4°C. Sonicated cells were incubated with antibody overnight at 4°C with rotation. Agarose beads were washed three times with modified buffer II/III. Washed beads were added to sonicated cells and incubated for 2 hours at 4°C with rotation. Bead-antibody-conjugated cells were washed two times with ice-cold low-salt washing buffer [20 mM tris-HCl (pH 8.1), 0.1% SDS, 1% Triton X-100, 2 mM EDTA pH 8.0, 150 mM NaCl], two times with ice-cold high-salt washing buffer [20 mM tris-HCl (pH 8.1), 0.1% SDS, 1% Triton X-100, 2 mM EDTA (pH 8.0), and 500 mM NaCl), two times with ice-cold LiCl washing buffer [10 mM tris-HCl (pH 8.1), 250 mM LiCl, 1% NP-40, 1 mM EDTA (pH 8.0), and 1% DOC], and one time with RT 1X TE buffer. DNA was eluted with elution buffer [10 mM tris-HCl (pH 8.0), 1 mM EDTA (pH 8.0), and 1% SDS] and reverse-cross-linked for 5 hours at 65°C. RNA and protein were removed using RNase A (BIOSESANG, R1007) and Proteinase K (NEB, Ipswich, MA, USA P8107S), respectively, and DNA was purified with UltraPure Phenol:Chloroform:Isoamyl Alcohol (25:24:1, v/v) (Thermo Fisher Scientific, 15593031) and the MinElute PCR purification kit (QIAGEN, Hilden, Germany, 28006).

ChIP-seq libraries were generated with the NEBNext End Repair Module (NEB, E6050L), NEBNext dA-Tailing Module (NEB, E6053L), Quick Ligation Kit (NEB, M2200L), NEBNext Multiplex Oligos for Illumina (NEB, E7335S, E7500S, and E7710S), and Phusion High-Fidelity DNA Polymerase (NEB, M0530L) according to the manufacturer’s protocol. ChIP-seq samples were sequenced on the Illumina NextSeq500 (Illumina, San Diego, CA, USA) with 75–base pair (bp) single-end reads.

### mRNA sequencing

Total RNA was prepared from MCF-7 cells after Q901 or TPT treatment using TRIzol (Invitrogen, 15596026) according to the manufacturer’s protocol. The mRNA sequencing (mRNA-seq) library was constructed using the MGIEasy RNA Directional Library Prep Kit (MGI, Shenzhen, China) according to the manufacturer’s instructions and sequenced on the MGISEQ-2000 (MGI) with 100-bp paired-end reads.

### fastGRO

fastGRO sample preparation was performed as previously described with minor modifications (*46*). After Q901 and TPT were treated, nuclei were isolated with ice-cold swelling buffer [10 mM tris-HCl (pH 7.5), 2 mM MgCl_2_, 3 mM CaCl_2_, and SUPERase·In RNase inhibitor (2 U/ml; Invitrogen, AM2696)], swelling buffer with glycerol [10 mM tris-HCl (pH 7.5), 2 mM MgCl_2_, 3 mM CaCl_2_, 10% glycerol, and SUPERase·In RNase inhibitor (2 U/ml)], and lysis buffer [10 mM tris-HCl (pH 7.5), 2 mM MgCl_2_, 3 mM CaCl_2_, 10% glycerol, 1% NP-40, and SUPERase·In RNase inhibitor (2 U/ml)]. Nuclei were stored in freeze buffer [50 mM tris-HCl (pH 8.0), 40% glycerol, 5 mM MgCl_2_, 0.1 mM EDTA (pH 8.0), and SUPERase·In RNase inhibitor (2 U/ml)] at −80°C. At the run-on step, nuclei were incubated with nuclear run-on buffer including 4-thio-uridine triphosphate (UTP) [10 mM tris-HCl (pH 8.0), 5 mM MgCl_2_, 1 mM dithiothreitol (DTT), 300 mM KCl, 500 μM ATP, 500 μM guanosine triphosphate (GTP), 2 μM cytidine triphosphate (CTP), 500 μM 4-S-UTP, 1% sarkosyl, and SUPERase·In RNase inhibitor] for 7 min. After total RNA precipitation, RNA was fragmented by sonication and biotinylated using the EZ-link HPDP-Biotin (Thermo Fisher Scientific, A35390). Biotinylated RNA was extracted using RNA precipitation and streptavidin-coupled magnetic beads (Dynabeads MyOne Streptavidin C1, Invitrogen, 65001).

Library preparation was performed following the qPRO-seq protocol (*47*). Following the ligation of the 3′ adapter, samples were treated with RppH (NEB, M0356S) and PNK (NEB, M0201S), and the 5′ adapter was ligated. RNA was purified using streptavidin-coupled magnetic beads and washed between incubation steps with high-salt buffer [50 mM tris-HCl (pH 7.4), 2 M NaCl, 0.5% Triton X-100, 1 mM EDTA (pH 8.0), and SUPERase·In RNase inhibitor (40 U/10 ml)] and low-salt buffer [50 mM tris-HCl (pH 7.4), 0.1% Triton X-100, 1 mM EDTA (pH 8.0), and SUPERase·In RNase inhibitor (40 U/10 ml)]. The biotinylated RNA was converted to DNA with a reverse-transcription step (Maxima H-reverse transcriptase, Thermo Fisher Scientific, EP0751) and amplified (Q5 High-Fidelity DNA Polymerase, NEB, M0491L) for sequencing. fastGRO samples were sequenced on NovaSeq 6000 (Illumina) with 100-bp paired-end reads.

### T-DXd serum stability assay

The serum stability of T-DXd was evaluated in male CD-1 mouse serum (Vital River Laboratory Animal Technology Co. Ltd., Beijing, China). Blood was collected via cardiac puncture under anesthesia, and serum was isolated by centrifugation (3000*g*, 10 min, 4°C), aliquoted, and stored at −80°C until use. T-DXd was spiked into the mouse serum at a final concentration of 100 μg/ml and incubated at 37°C for 0, 1, 4, 7, 14, and 21 days. Buffer controls were prepared by incubating T-DXd (100 μg/ml) in PBS (Gibco, 2661454) containing 1% BSA (BBI J711BA007). High-quality control (HQC) samples were prepared by spiking T-DXd into mouse serum at the same concentration. Samples were processed using the High Capacity Magne Streptavidin Beads (Promega, 576739), charged with Goat Anti-human Biotinylated IgG (Promega, 571395). Serum samples (20 μl) were diluted with 130 μl of bind/wash buffer (200 mM NaCl in PBS, prepared with NaCl from Bidepharm, EKZ867, and PBS from Gibco, 2661454) and incubated with the beads at 1100 rpm for 120 min at RT. Beads were washed twice with bind/wash buffer, once with PBS, and once with purified water (ELGA, NA). For elution and reduction, 50 μl of elution buffer (0.1% trifluoroacetic acid in water; J&K, L910X51) was added, and the mixture was shaken at 1100 rpm for 30 min at RT. The eluate was neutralized with 5 μl of neutralization buffer (1 M ammonium bicarbonate; LingFeng, 20231115) and 5 μl of DTT (TCI, OJWPT-DA). Samples were shaken for another 30 min at RT and centrifuged at 3220*g* for 5 min. Liquid chromatography–tandem mass spectrometry (LC-MS/MS) analysis was performed using a Waters VION QTof mass spectrometer operated in sensitivity mode (ESI+, *m/z* 800 to 2500). The source temperature was set to 150°C, with a desolvation temperature of 600°C, a desolvation gas flow at 700 liters/hour, and a cone gas flow at 50 liters/hour. The capillary voltage was 2.75 kV, the sample cone voltage was 25 V, and the source offset voltage was 40 V. LockSpray was applied using *m/z* 785.8421 as the reference. Chromatographic separation was performed on an Agilent PLRP-S column (1 mm by 50 mm, 5 μm, 1000 Å; Agilent Technologies, AT-PL1312-1502) at 60°C with a flow rate of 0.3 ml/min. The mobile phases consisted of 0.1% formic acid (Thermo Fisher Scientific, A117) in water (A) and 0.1% formic acid in acetonitrile (Merck, 1.000.30.4008) (B). The gradient elution was programmed from 10 to 85% B over 8 min, followed by reequilibration to initial conditions by 12 min. The DAR values of T-DXd over time were measured. This study was performed in duplicate. Data represent mean ± SD.

### γH2AX IF assay

For in vivo assessment of DNA damage, HCT116 xenograft tumors were established by subcutaneous inoculation of 5 × 10^6^ cells in 0.1 ml of PBS into the right upper flank of female BALB/c nude mice (6 to 8 weeks old). When tumors reached ∼120 mm^3^, mice were randomized into four groups (*n* = 9 per group) and treated with vehicle, Q901 (10 mg/kg, intraperitoneally once daily), T-DXd (10 mg/kg, single intravenous injection), or their combination. Tumor samples were collected at day 7 and fixed in formalin for paraffin embedding. Sections (4 μm) were prepared using a Leica HistoCore BIOCUT microtome and baked at 60°C for 60 min. Deparaffinization, rehydration, and IF staining were performed on a Leica Bond RX automated staining platform. Antigen retrieval was performed using the Bond Epitope Retrieval Solution 1 (Leica, AR9961) for 20 min. Sections were incubated with a phospho-histone H2A.X (Ser^139^) rabbit monoclonal antibody (1:500; Cell Signaling Technology, 9718) and detected using an Opal 620 detection kit (1:100; Akoya Biosciences, NEL821001KT). Slides were mounted with the ProLong Diamond Antifade Mountant (Invitrogen, P36961) and scanned using a Leica MICA imaging system. Images were analyzed using the HALO software (Indica Labs) with H-score calculated on the basis of foci counts per cell, with minimum copies/cell thresholds of 1, 4, 8, and 12 for scores of 1+, 2+, 3+, and 4+, respectively. Representative images were cropped using ImageJ (1.54), and scale bars represent 20 μm.

### Cell line–derived xenograft models

BALB/c nude mice were purchased from the Shanghai SIPPR/BK Laboratory Animal Co. Ltd. (Shanghai, China). For in vivo efficacy studies, HCT116 cells or H292 cells were resuspended in PBS and Matrigel (1:1) and implanted subcutaneously. When tumors reached an average size of 117 mm^3^ (HCT116) or 140 mm^3^ (H292), mice were randomized into four groups (*n* = 8 per group) and treated with Q901 (3 or 10 mg/kg, intraperitoneally once daily), T-DXd (10 mg/kg, single intravenous administration on day 0), SG (3 mg/kg, single intravenous administration on days 1 and 8), or a combination thereof. Tumor volumes were measured twice weekly after randomization in two dimensions, using a caliper. The TGI was calculated for each group using the formula: TGI (%) = [1 − (*T*_i_ − *T*_0_)/(*V*_i_ − *V*_0_)] × 100, where *T*_i_ is the average tumor volume of the treatment group on the given day, *T*_0_ is the average tumor volume of the treatment group on the day when the treatment was started, *V*_i_ is the average tumor volume of the vehicle control group on the same day with *T*_i_, and *V*_0_ is the average tumor volume of the vehicle group on the day of treatment start. Statistical significance was calculated using a two-way analysis of variance (ANOVA) followed by Tukey’s multiple comparison test in GraphPad Prism 10.4.1.

### SynergyFinder

SynergyFinder was performed by Oncolines (Oss, Netherlands). Cells were treated with Q901, T-DXd, or SG for 72 hours, and cell viability was assessed to determine the IC_50_ of Q901, T-DXd, or SG in the respective cell lines. Because HCT116 cells were insensitive to T-DXd, T-DXd (10 μg/ml) was mixed with various concentrations of Q901 in HCT116 cells. In H292 cells, which are sensitive to SG, stock solutions of Q901 and SG were diluted to their respective IC_50_ concentrations, as determined in single-agent experiments, and then mixed at a 1:4 ratio (Q901:SG = 1:4). The mixtures, Q901, and SG were further diluted to generate a seven-point dose-response series in duplicate. For single agents, the final assay concentration range is from 10 to 0.01 times their IC_50_ (10 and 0.01 equiv). Dose-response curves were fitted using nonlinear regression with a sigmoidal dose-response model in GraphPad Prism 10.4.1 or XL-fit 5. The absolute IC_5__0_ values were determined from the dose-response curves generated in XL-fit 5 using the four-parameter logistic equation: fit = *A* + ((*B* − *A*)/{1 + [(*C*/x)*^D^*]}), where *A* and *B* represent the bottom and top plateaus of the curve, *C* is the IC_5__0_ value, *x* is the compound concentration, and *D* is the Hill slope.

### Data analysis

#### ChIP-seq analysis

ChIP-seq data were trimmed with Trim Galore (0.6.7) (https://github.com/FelixKrueger/TrimGalore) and aligned to hg38 (v36) using Bowtie2 (2.4.5) (https://github.com/BenLangmead/bowtie2). The bigWig files were generated by HOMER (v4.11) (http://homer.ucsd.edu/homer/) makeUCSCfile and visualized with the UCSC genome browser. For pan RNAPII, Ser5P, and Ser2P ChIP-seq, read counting was generated by HOMER analyzeRepeats.pl and DESeq2 (1.40.0) (https://bioconductor.org/packages/DESeq2/). For CDK7, MYC, and E2F1 ChIP-seq, peakcalling was performed with MACS3 (3.0.0b1) (https://github.com/macs3-project/MACS) and IDR (2.0.3) (https://github.com/hbctraining/Intro-to-ChIPseq).

#### mRNA-seq analysis

mRNA-seq data were trimmed with Trim Galore and aligned to hg38 (v36) using STAR (2.7.8a_2021-03-08) (https://github.com/alexdobin/STAR). The bigWig files were generated by deepTools bamCoverage (3.5.1) and visualized with the UCSC genome browser. The expression level was calculated with RSEM rsem-calculate-expression (v1.3.1) (https://github.com/deweylab/RSEM) and DESeq2.

#### fastGRO analysis

fastGRO data were trimmed with fastp (0.23.4) (https://github.com/OpenGene/fastp), and rRNA was removed. The trimmed reads were aligned to hg38 using Bowtie2 and deduplicated with UMI-tools (1.1.4) (https://github.com/CGATOxford/UMI-tools). The bigWig files were generated by deepTools (https://github.com/deeptools/deepTools) bamCoverage (3.5.1) and visualized with the UCSC genome browser.

#### Average plot and heatmap

Average plot and heatmap were generated by deepTools computeMatrix and plotHeatmap (3.5.1).

#### mRNA half-life analysis

mRNA half-life values were obtained from the published dataset by Lugowski *et al.* (*48*). For down-regulated genes identified at each time point (1, 4, and 24 hours) in our Q901 treatment experiments, we extracted the corresponding mRNA half-life values from this dataset and plotted them against the log_2_ fold changes from our mRNA-seq analysis.

#### Pathway analysis

GSEA was performed using the R package fgsea (1.26.0) (https://github.com/alserglab/fgsea). The datasets were used from MSigDB (v2023.1) (https://gsea-msigdb.org/gsea/msigdb). GO analysis of enhancer target genes and hallmark pathway enrichment analysis of intersecting genes from E2F1 and MYC cluster1 were performed using the Metascape (https://metascape.org/).

#### Enhancer calling

The enhancers were defined using H3K27ac ChIP-seq from GSE62229 (*32*). ChIP-seq data were processed same as CDK7, MYC, and E2F1 ChIP-seq except for the peakcalling step (MACS3 option: --broad). ROSE (https://github.com/stjude/ROSE) was used to define SEs with H3K27ac peaks (-s 12500 -t 2000). The defined enhancers that did not overlap with SE were defined as TEs. CDK7-bound enhancers were defined as enhancers that overlapped with peaks showing increased CDK7 binding upon Q901 treatment compared to DMSO. CDK7-nonbound enhancers were defined as the remaining enhancers after excluding CDK7-bound enhancers from the total enhancer set. The target protein-coding genes of SE and TE were defined as protein-coding genes located within a 50-kb region from the start or end of SE/TE. To ensure that the four groups did not overlap, priority was given to SE CDK7-bound. For target genes containing at least one SE, those with at least one CDK7-bound SE were classified as SE CDK7-bound target genes, whereas those without any CDK7-bound SE were classified as SE CDK7-nonbound target genes. The same classification criteria were applied to TE target genes that did not contain any SE.

### Statistical analysis

Statistical analyses were performed using GraphPad Prism 10.4.1. Statistical approaches are specified for each experiment, and only *P* value ≤ 0.05 are considered significant.
