# Supplementary material for: Sensitizing tumor response to topoisomerase I antibody-drug conjugate by selective CDK7 inhibition
Source: Sci Adv. 2026 Sep 30;12(40):eaeb4023. doi: 10.1126/sciadv.aeb4023 (PMC13626057; doi:10.1126/sciadv.aeb4023)
Supplement: Supplementary file 1 [file sciadv.aeb4023_sm.pdf]

Science Advances
AAAS

Supplementary Materials for

Sensitizing tumor response to topoisomerase I antibody-drug conjugate by selective CDK7 inhibition

Hyerim Jung *et al.*

Corresponding author: Kiyean Nam, knam@qurient.com; Tae-Kyung Kim, tkkim@postech.ac.kr

Sci. Adv. **12**, eaeb4023 (2026)
DOI: 10.1126/sciadv.aeb4023

This PDF file includes:

Figs. S1 to S14
Table S1

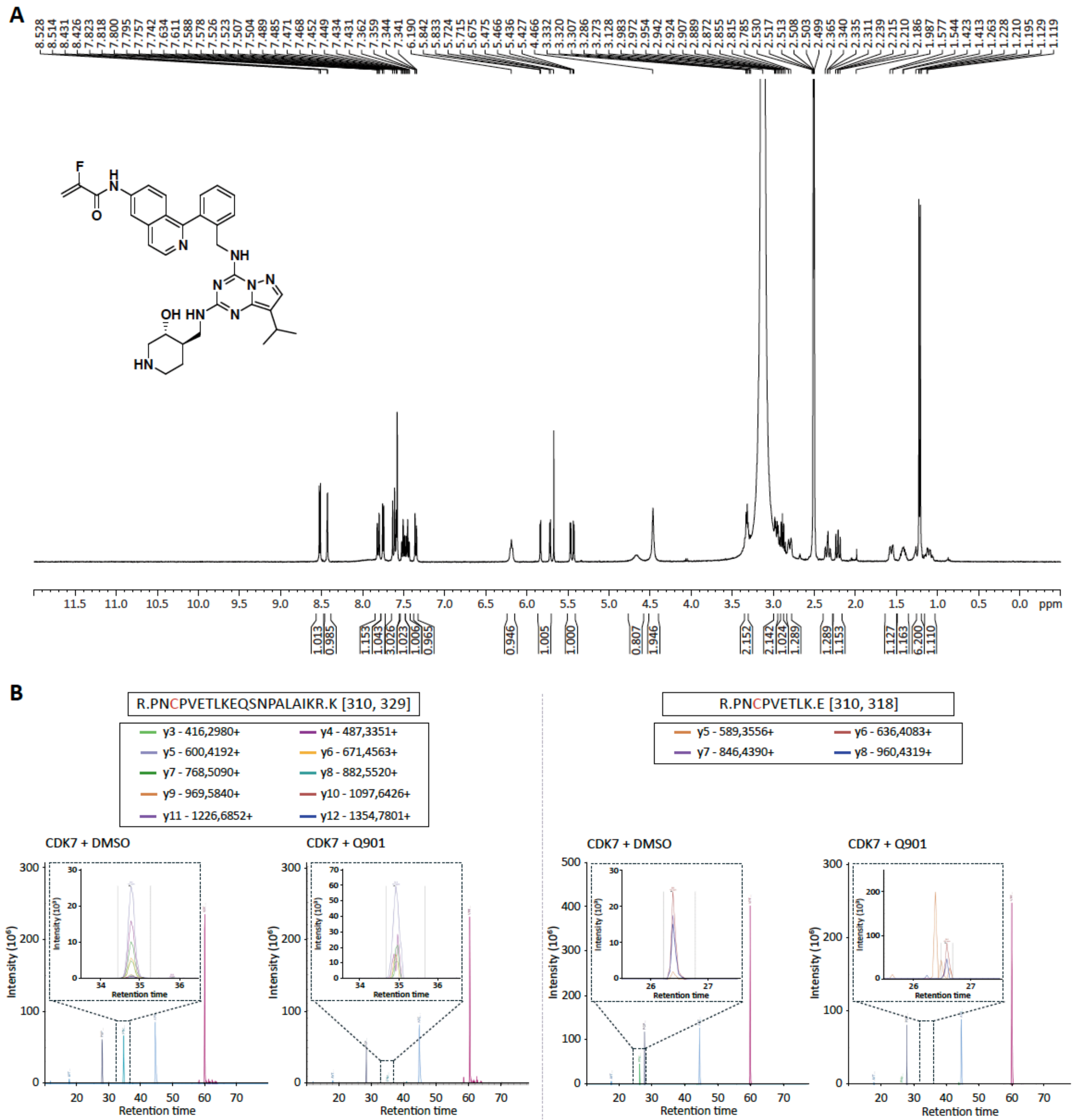

Fig. S1. Biochemical and proteomic validation of Q901 specificity.
(**A**) ^{1}H NMR spectrum of Q901 was acquired using variable temperature (VT) NMR in DMSO-d_6. (**B**) Targeted proteomics analysis to determine the Q901 binding sites on CDK7. The recombinant CAK trimeric complex was incubated with Q901 or DMSO, followed by protease digestion and peptide mapping via LC-MS/MS. Chromatograms show peptide fragments generated by Arg-C digestion (left) and Arg-C/Trypsin digestion (right). The expanded boxes highlight the peak of C312 containing peptides, which are reduced following Q901 treatment, indicating covalent modification at this site.

A

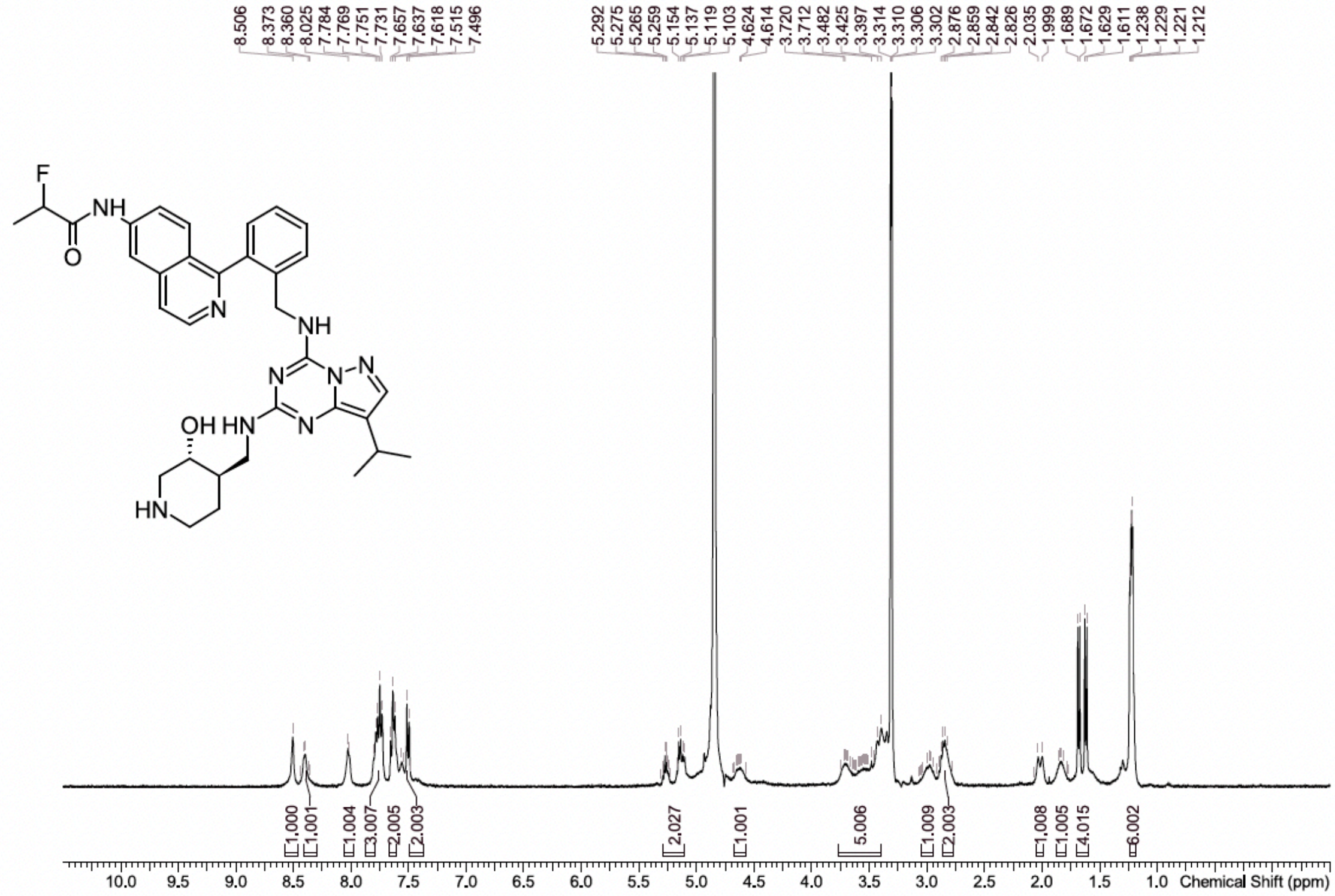

B

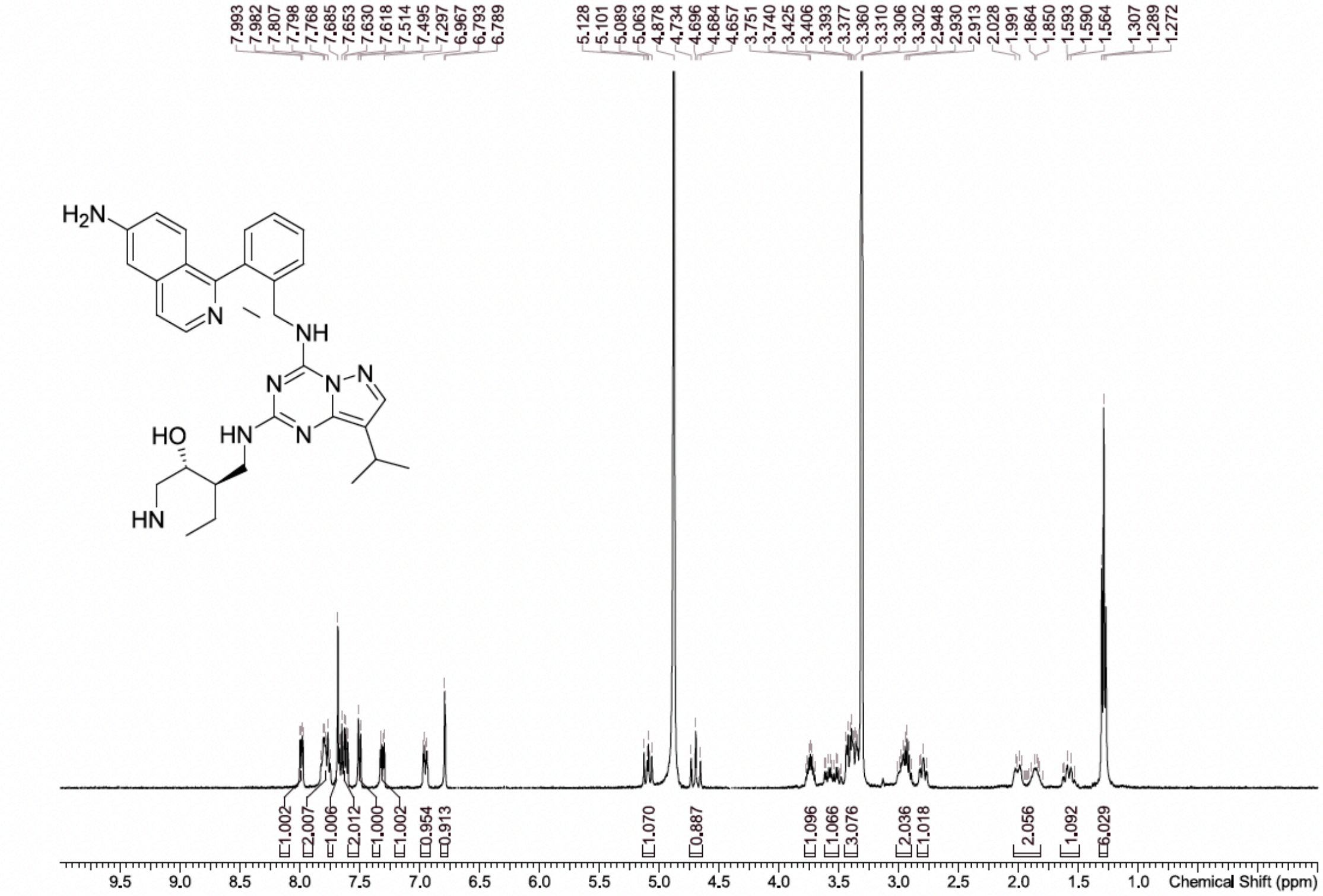

Fig. S2. Biochemical and proteomic validation of non-covalent CDK7 inhibitor analogues specificity.

^{1}H NMR spectrum of QS0551546 (Q901R) (A) and QS0551508 (B) was acquired using variable temperature (VT) NMR in DMSO-d_6.

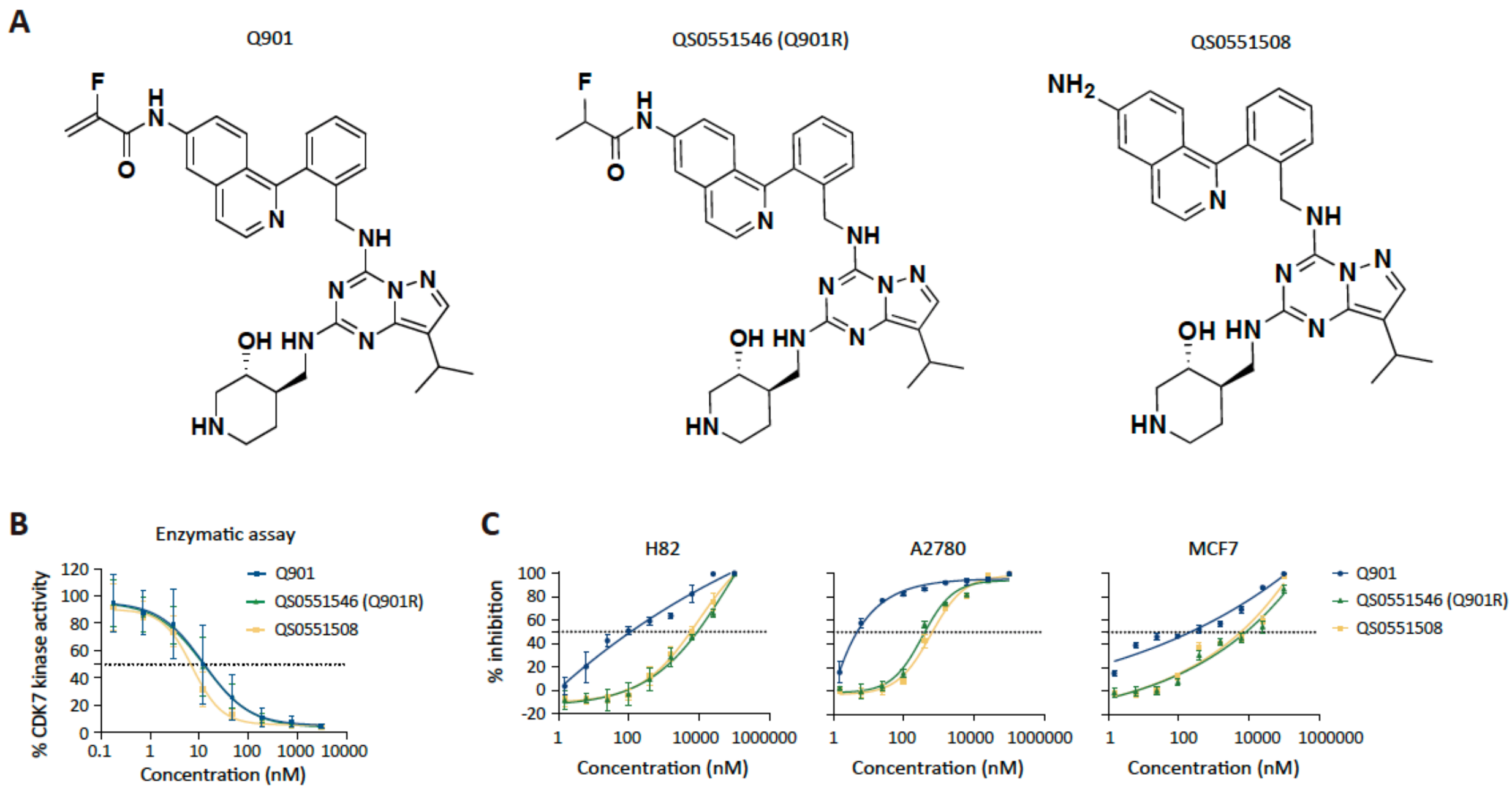

Fig. S3. Non-covalent CDK7 inhibitors.
(**A**) Chemical structure of Q901, QS0551546 (Q901R) and QS0551508. (**B**) Enzymatic inhibition of CDK7 by Q901 and related compounds measured using a LANCE Ultra kinase assay. Recombinant CDK7 activity was assessed in the presence of Q901, QS0551546 (Q901R), and QS0551508 across an 8-point serial dilution (top concentration: 3000 nM, 1:4 dilution). Data are presented as % kinase activity relative to control (n = 3). (**C**) Cellular inhibition of Q901 and related compounds in H82, A2780, and MCF7 cells measured using a CTG assay. Cells were treated with Q901, QS0551546 (Q901R), and QS0551508 across a 9-point serial dilution (top concentration: 100 μM, 1:4 dilution) for 72 h. Data are presented as % inhibition relative to control (n = 3).

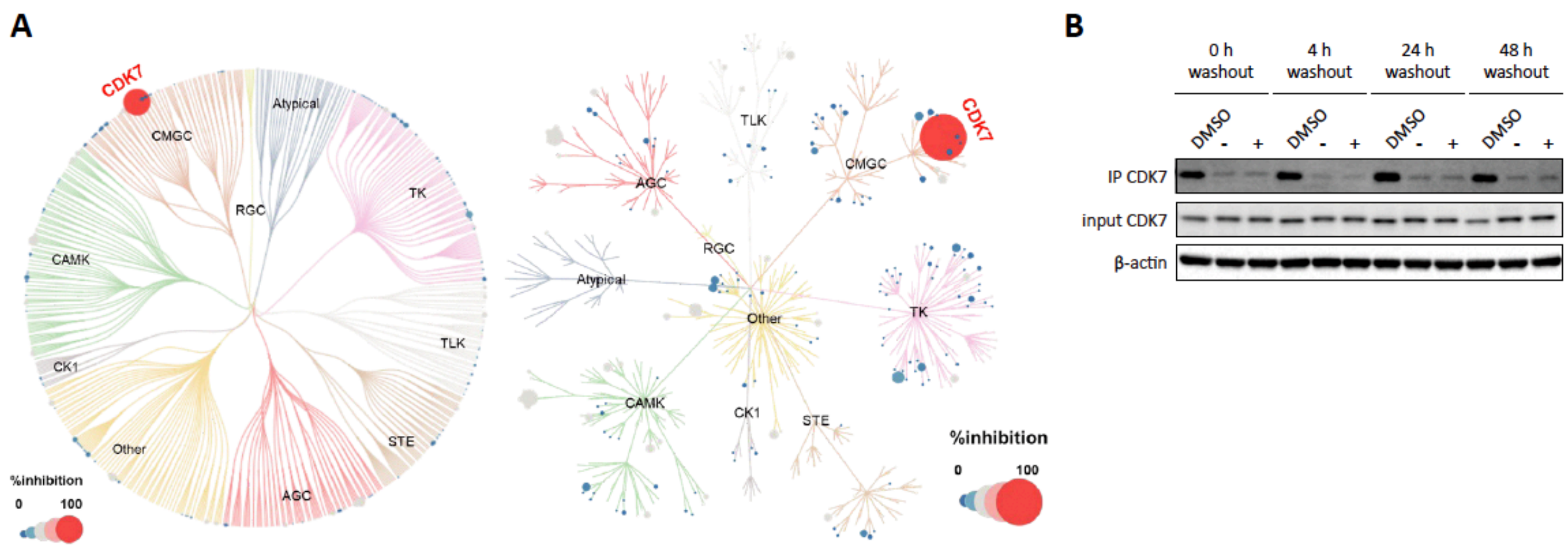

Fig. S4. Kinome-wide selectivity and sustained target engagement of Q901.
(**A**) Kinome-wide selectivity of Q901 visualized using Coral (Metz et al., 2018) based on the kinase profiling dataset shown in Fig. 1C. Node size and color represent the degree of kinase inhibition, illustrating the selective targeting of CDK7. (**B**) Representative Western blot images from the pulse-chase assay described in Fig. 1F. A2780 cells were treated with 6 nM Q901 for 4 h and then divided into two groups. One group (- wash out) remained in the Q901-containing medium for continuous incubation, while the other group (+ wash out) underwent a drug washout, where the medium was completely removed and replaced with fresh drug-free medium before further incubation for the indicated times. Bio-QS-labeled CDK7 was immunoprecipitated using streptavidin agarose beads (SA), and the levels of free CDK7 were analyzed by immunoblotting. These images in this figure were quantified in Fig. 1F.

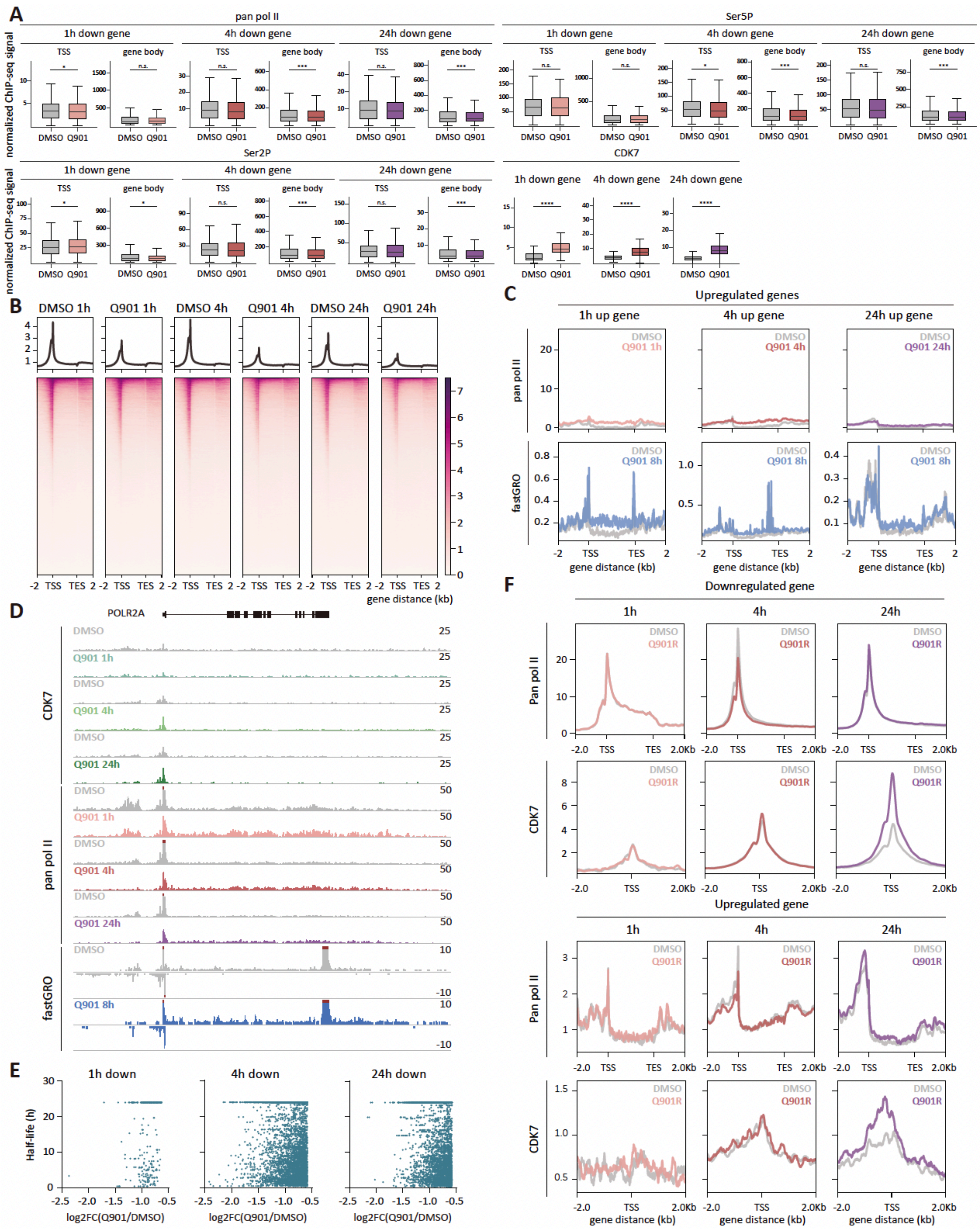

Fig. S5. Global and gene-specific changes in RNAPII occupancy following Q901 treatment.

(**A**) Box plot of pan pol II, Ser5P, Ser2P and CDK7 ChIP-seq for downregulated genes in Fig. 2C and D. TSS (−50 to +300 bp) and gene body (+300 bp to TES) were counted and outliers (>99th percentile) were excluded (*: $p < 0.05$, ***: $p < 0.001$, ****: $p < 0.0001$ by Wilcoxon

signed-rank test (Shapiro–Wilk test, $p < 0.05$)). (**B**) Heatmap of pan RNAPII ChIP-seq for all annotated genes ($n = 2$, p-value ≤ 0.05). (**C**) Average ChIP-seq plots of pan RNAPII and fastGRO for upregulated genes across various time points following Q901 treatment. Pan pol II was presented on the same scale as those for downregulated genes in Fig. 2D. (**D**) Track image of POLR2A, an example of an upregulated gene by Q901 treatment. (**E**) Scatter plots showing the correlation between the mRNA half-lives of downregulated genes and log2FC values of pan RNAPII ChIP-seq for the corresponding genes. (**F**) Average pan pol II and CDK7 plot of downregulated and upregulated genes using at Fig. 2C and D treated with QS0551546 (Q901R) for 1, 4, and 24h.

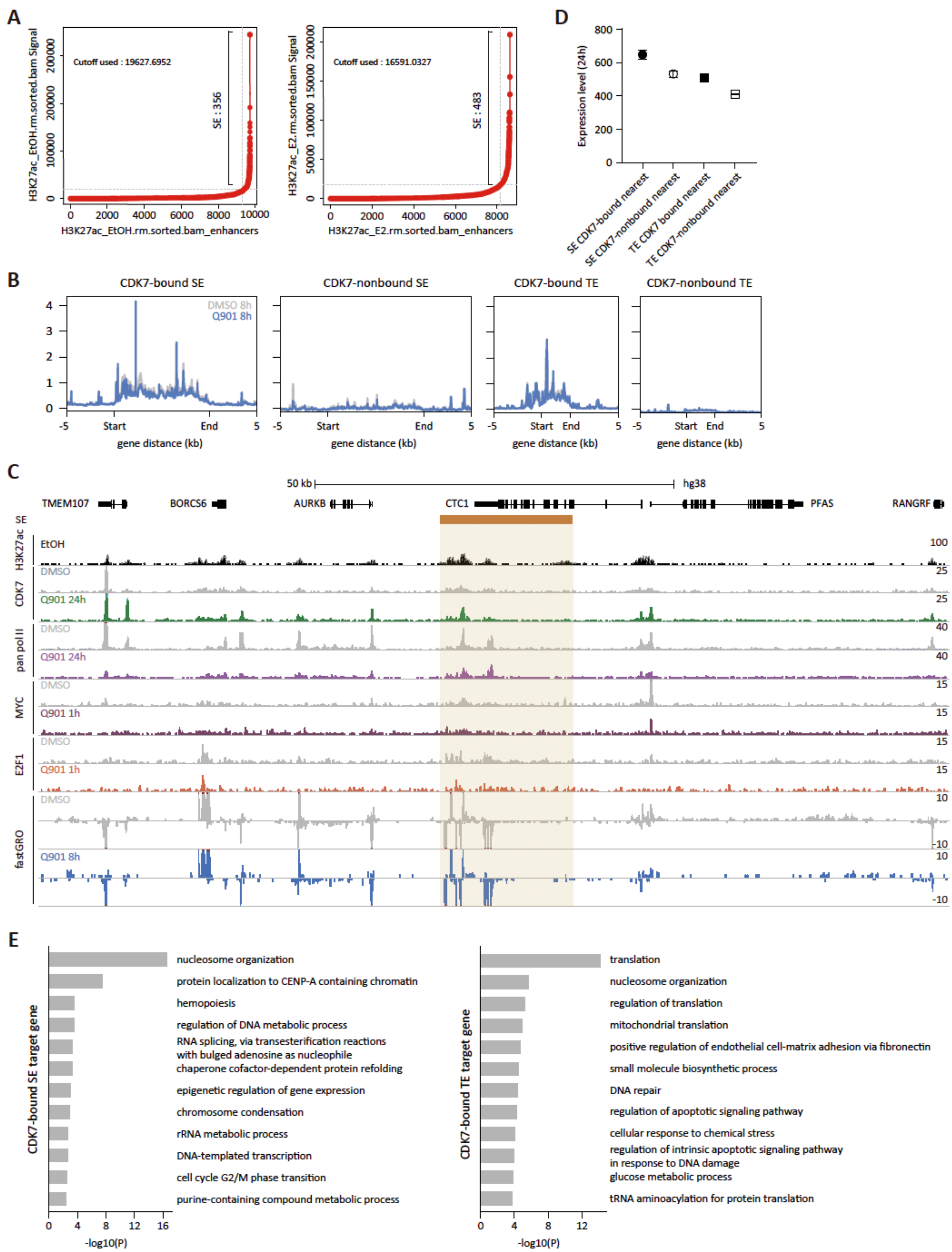

Fig. S6. Transcription profiles of enhancer target genes.
(**A**) The results of SE calling using the ROSE program with H3K27ac ChIP-seq (GSE62229). (**B**) Average fastGRO signals of four enhancer groups. (**C**) Track images showing ChIP-seq

signals for H3K27ac, CDK7, pan RNAPII, MYC, and E2F1, along with fastGRO, at a representative CDK7-bound SE region (highlighted in yellow) and it associated target genes. (**D**) Expression levels of enhancer target genes (pan RNAPII ChIP-seq; n = 2, DMSO 1h treatment condition, data represent mean ± SEM). (**E**) GO analysis results of target genes regulated by CDK7-bound SE and CDK7-bound TE.

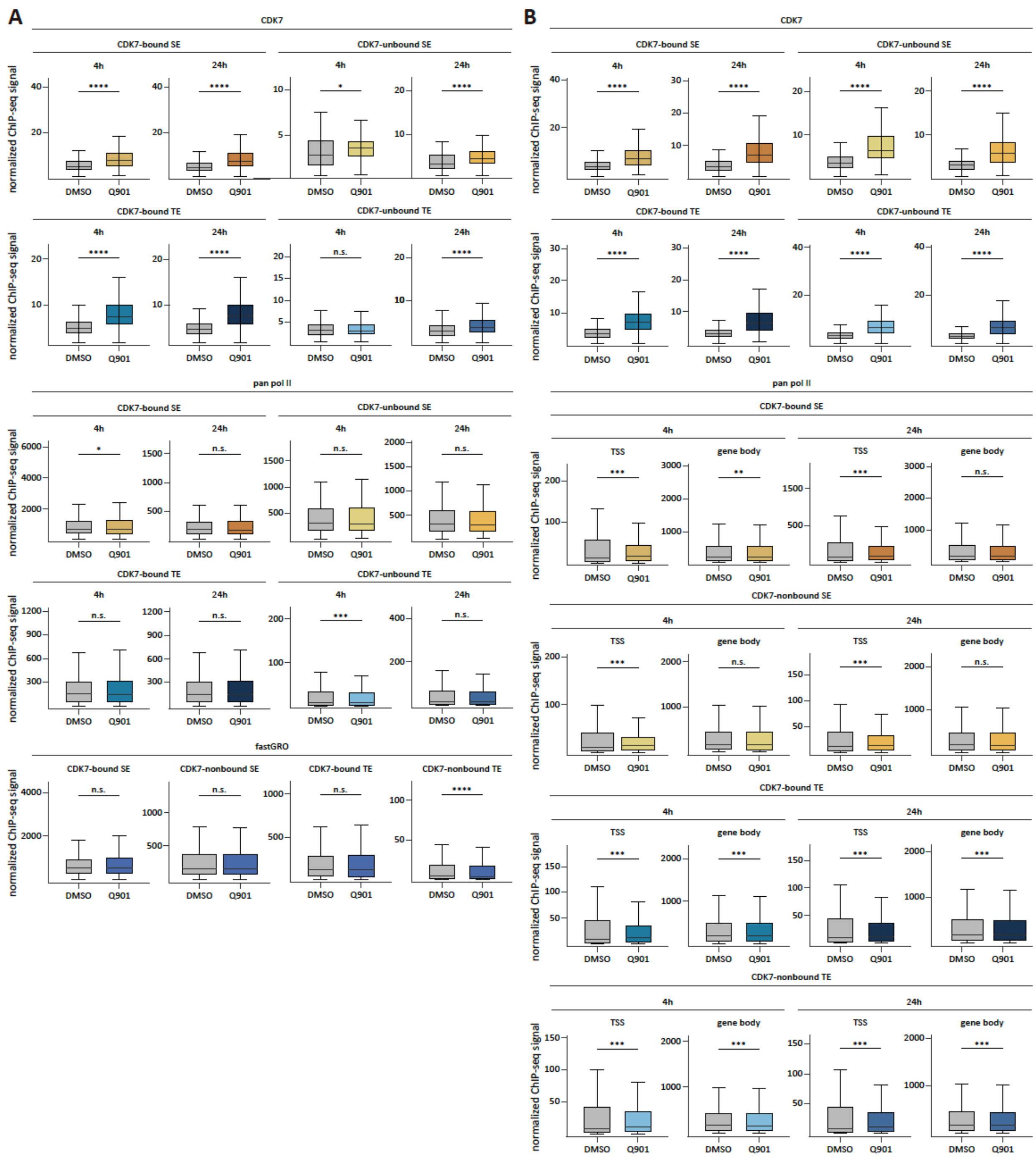

Fig. S7. Boxplot of enhancer and target gene regions.
(**A**) Box plot of CDK7, pan pol II ChIP-seq and fastGRO for enhancer regions in Fig. 3A. Outliers (>99th percentile) were excluded (*: $p < 0.05$, ***: $p < 0.001$, ****: $p < 0.0001$ by Wilcoxon signed-rank test (Shapiro–Wilk test, $p < 0.05$)). (**B**) Box plot of CDK7, and pan pol II ChIP-seq for enhancer target gene regions in Fig. 3B. TSS (−50 to +300 bp) and gene body (+300 bp to TES) were counted and outliers (>99th percentile) were excluded (**: $p < 0.01$, ***: $p < 0.001$, ****: $p < 0.0001$ by Wilcoxon signed-rank test (Shapiro–Wilk test, $p < 0.05$)).

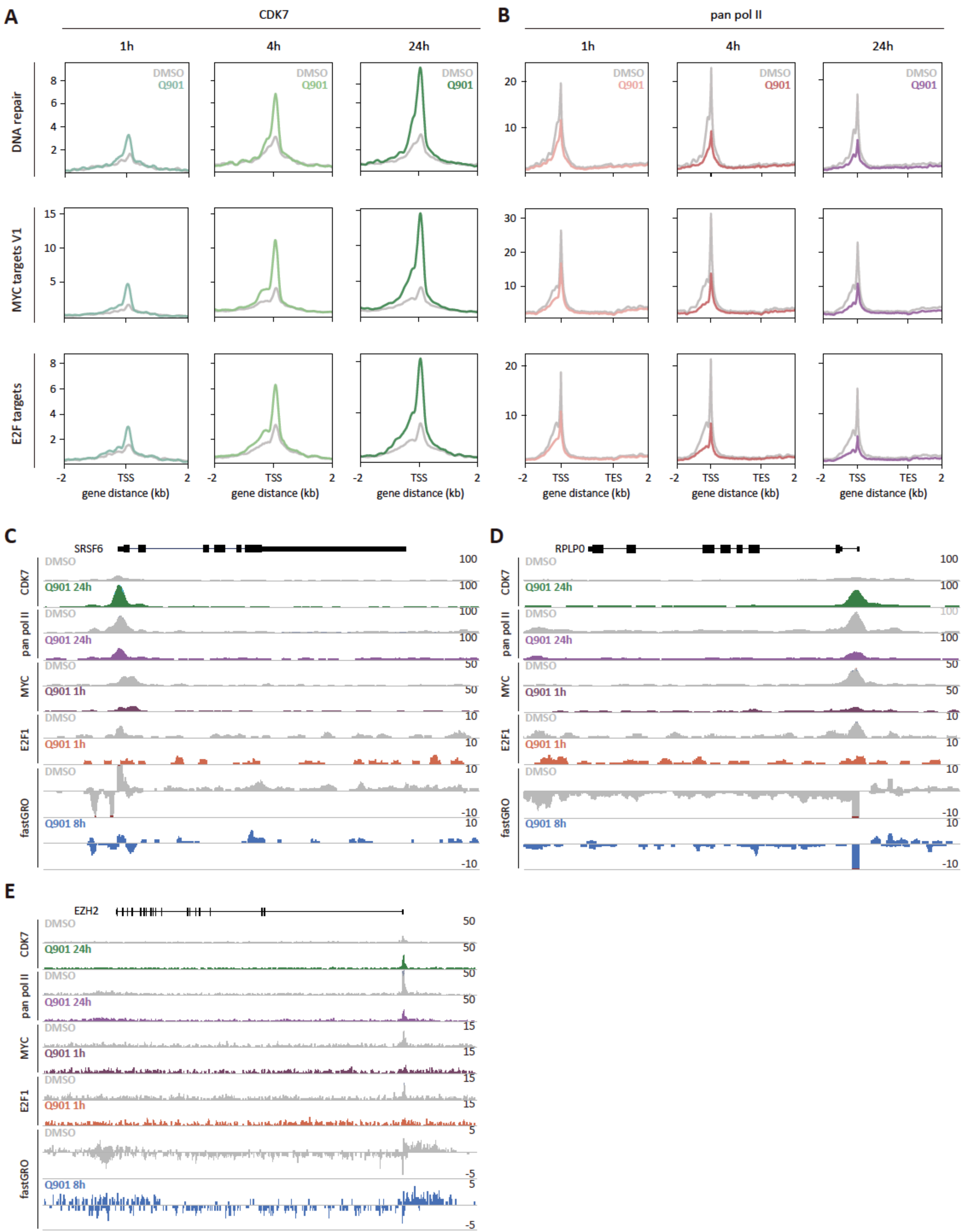

Fig. S8. CDK7 and RNAPII ChIP-seq signal at DNA Repair, MYC, and E2F target genes. (**A** and **B**) Average ChIP-seq signal plots of CDK7 (A) and pan RNAPII (B) for gene sets related to DNA repair, MYC targets V1, and E2F targets. (**C**) Track image of SRSF6 gene, a representative gene from the DNA repair pathway. (**D**) Track image of RPLP0 gene, a

representative gene from the MYC targets V1 pathway. (**E**) Track image of EZH2 gene, a representative gene from the E2F targets pathway.

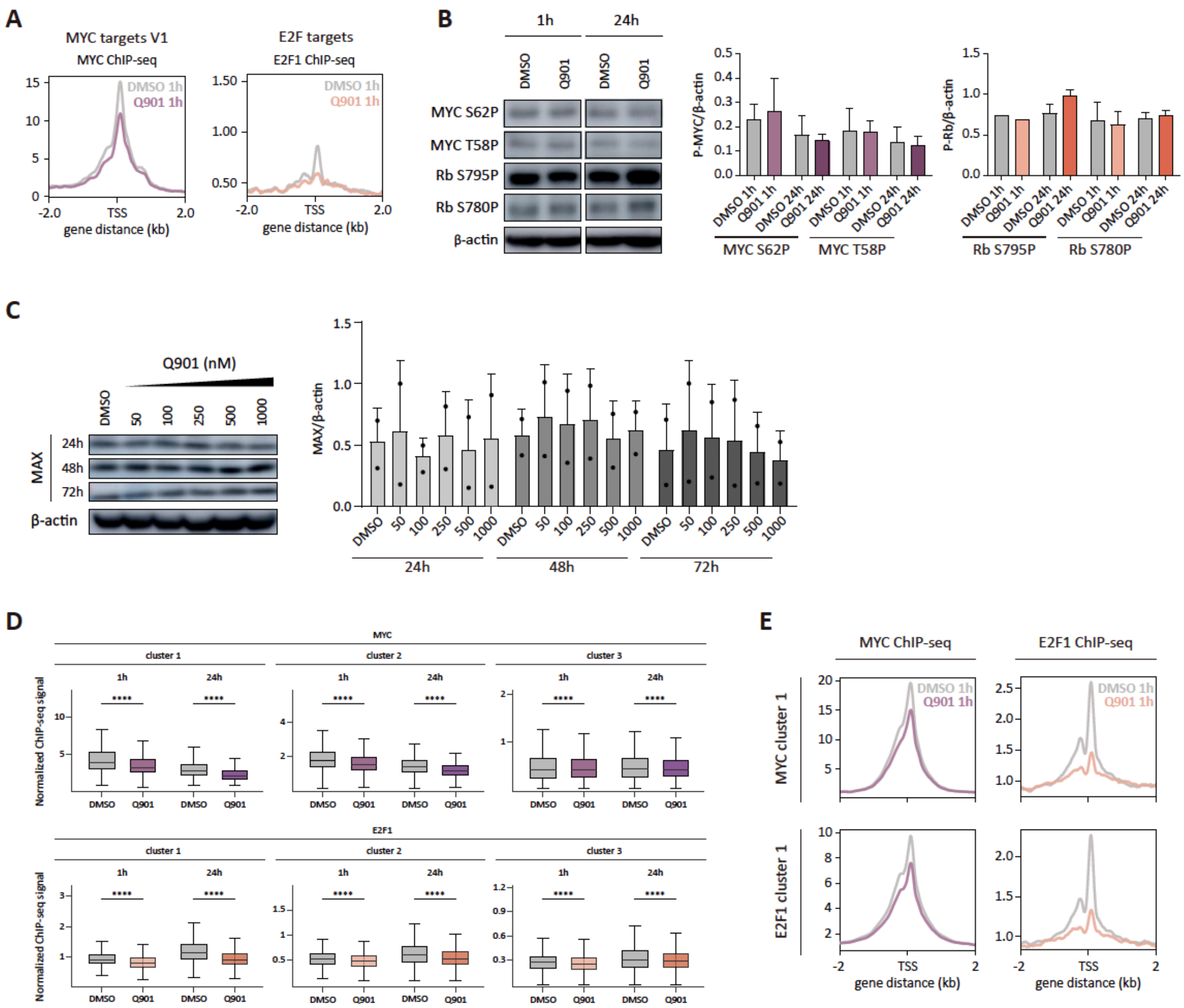

Fig. S9. Expression levels of MYC and E2F1 activity-related genes.
(**A**) Average MYC and E2F1 ChIP-seq signal plot of MYC targets V1 gene set after 1h Q901 treatment (n = 2). (**B**) Western blot data of phospho-MYC and phospho-Rb after Q901 treatment (n = 2 or 3; two-way ANOVA followed by Šidák's multiple comparison test, data represent mean ± SD). (**C**) Western blot data of MAX expression after Q901 treatment (n = 2; two-way ANOVA followed by Šidák's multiple comparison test, data represent mean ± SD). (**D**) Box plot of MYC and E2F1 ChIP-seq for MYC or E2F1 clustered genes in Fig. 4E. Outliers (>99th percentile) were excluded (****: $p < 0.0001$ by Wilcoxon signed-rank test). (**E**) Average plots of MYC and E2F1 ChIP-seq signals for MYC cluster 1 and E2F1 cluster 1 genes after 1h Q901 treatment.

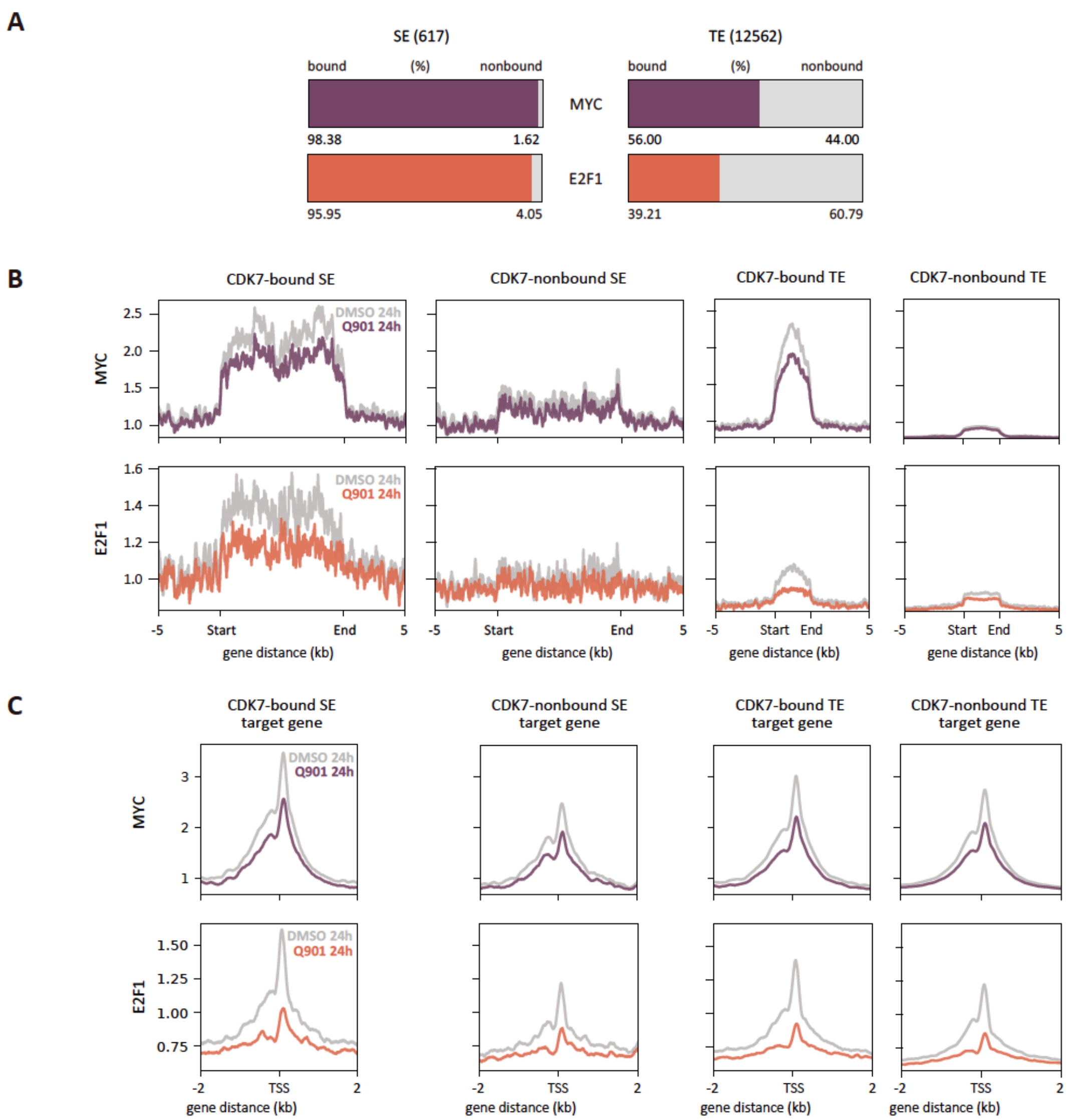

Fig. S10. MYC and E2F1 binding profiles across enhancer regions and their target genes. (**A**) Box plot showing the ratio of MYC/E2F1-bound and non-bound regions in SEs, TEs. (**B**) Average plots of MYC and E2F1 ChIP-seq signals across the four enhancer groups. (**C**) Average plots of MYC and E2F1 ChIP-seq signals across the four groups of enhancer target protein-coding genes.

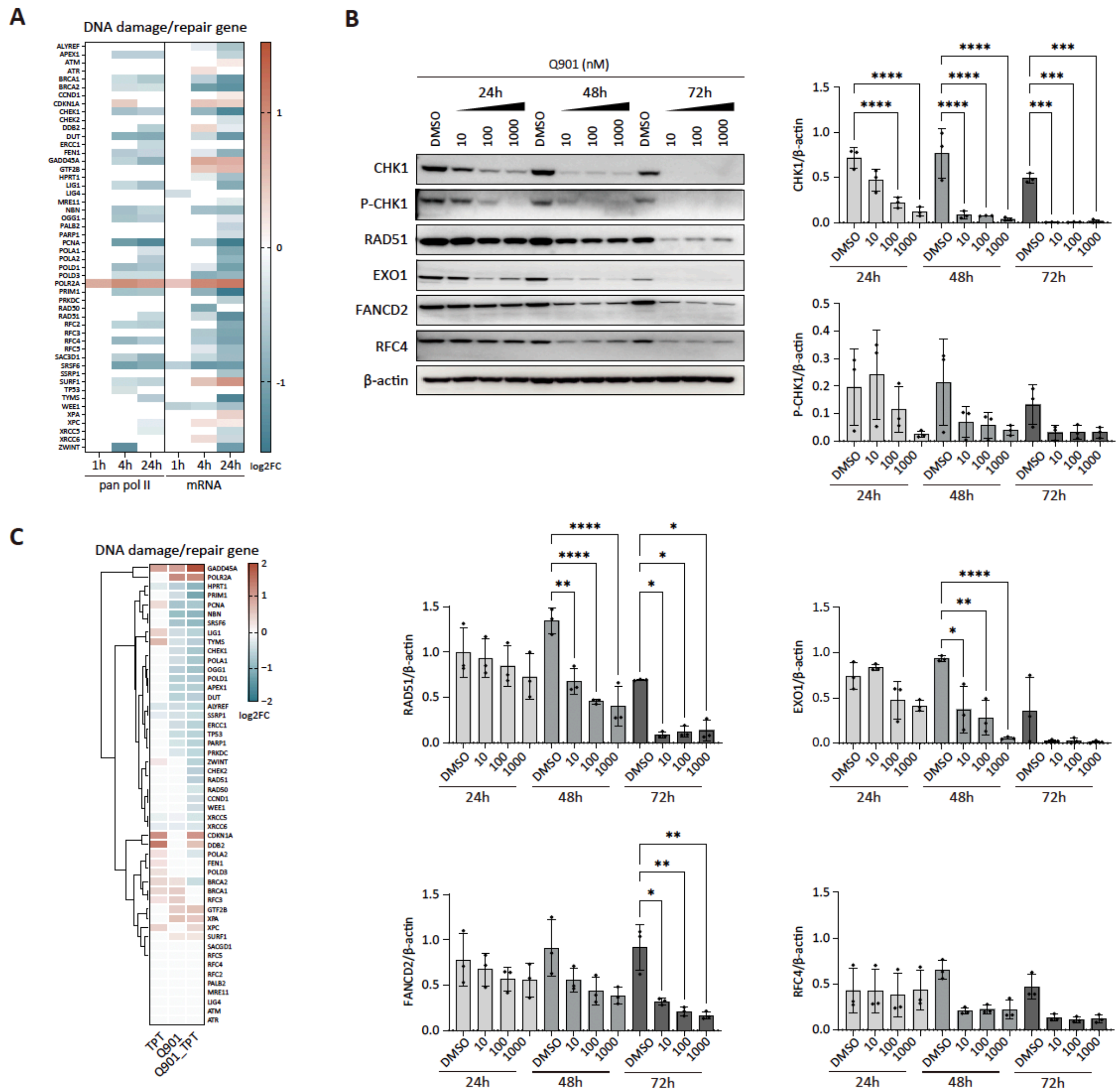

Fig. S11. DDR pathway.

(**A**) Heatmap showing log2FC values of DNA damage/repair genes expression from pan RNAPII ChIP-seq and mRNA-seq data (n = 3, FDR ≤ 0.1). (**B**) Western blot data of genes related with DDR pathway (n = 3, *: $p < 0.05$, **: $p < 0.01$, ***: $p < 0.001$, ****: $p < 0.0001$ by ordinary one-way ANOVA followed by Šídák's multiple comparisons test). (**C**) Heatmap showing log2FC values of DNA damage/repair genes expression from mRNA-seq data treated with TPT, Q901 and TPT+Q901 (n = 2, p-value ≤ 0.05). Columns were clustered by hierarchical clustering using Euclidean distance and complete linkage.

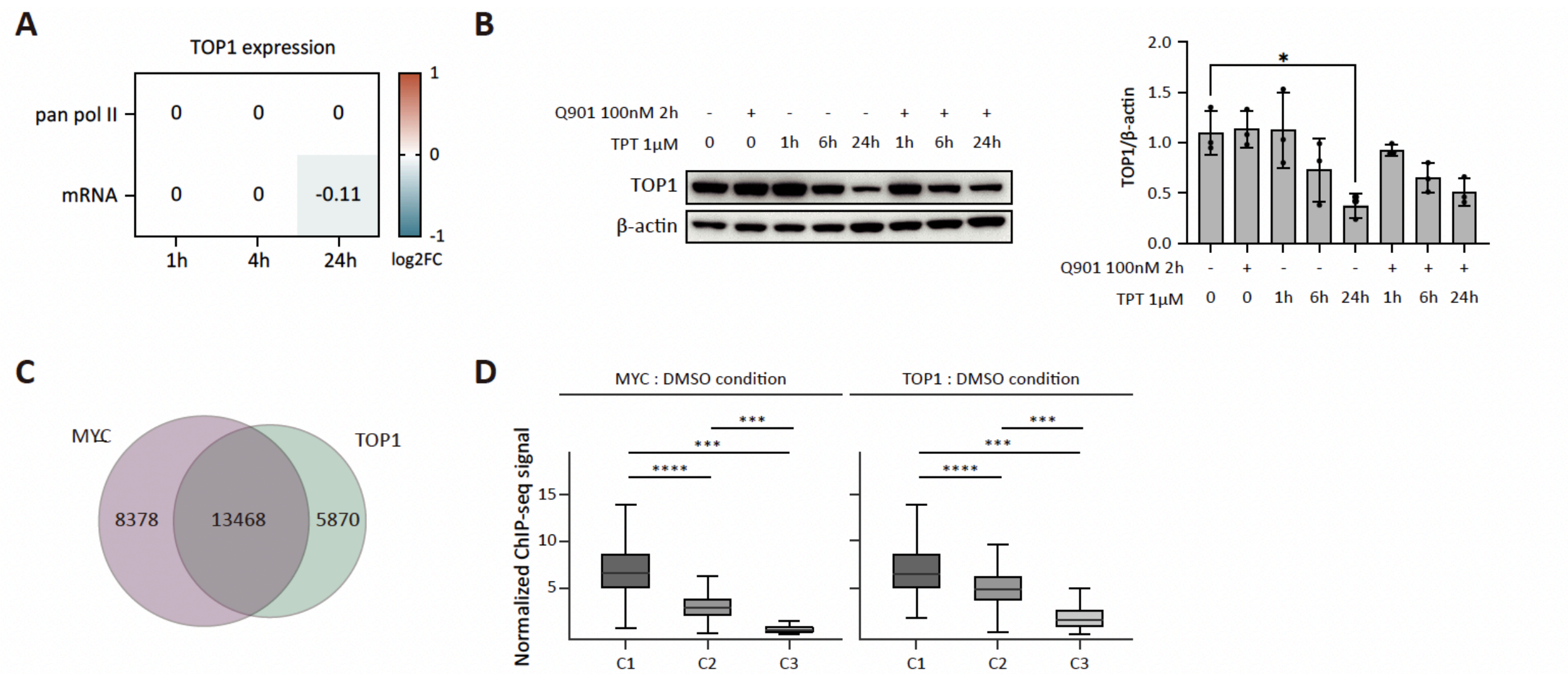

Fig. S12. Transcriptional and protein-level regulation of TOP1 in response to Q901 and TPT treatments.

(**A**) Heatmap displays log2FC (Q901/DMSO) from pan RNAPII ChIP-seq and mRNA-seq data for TOP1. (**B**) MCF-7 cells were pretreated with Q901 or DMSO for 2 h, followed by treatment with TPT for the indicated time points. Cell lysates were subjected to western blot assay using anti-TOP1 antibody (upper). Bar graph (bottom) shows quantification of TOP1 normalized to β-actin (n = 3; two-way ANOVA followed by Šidák's multiple comparison; data represent mean ± SD). (**C**)Venn diagram shows the overlap between MYC-bound and TOP1-bound genes. (**D**) Box plot of MYC and TOP1 clustering in Fig. 5A (***: p < 0.001, ****: p < 0.0001 by Kruskal-Wallis test with Dunn's post-hoc test (Benjamini-Hochberg correction)).

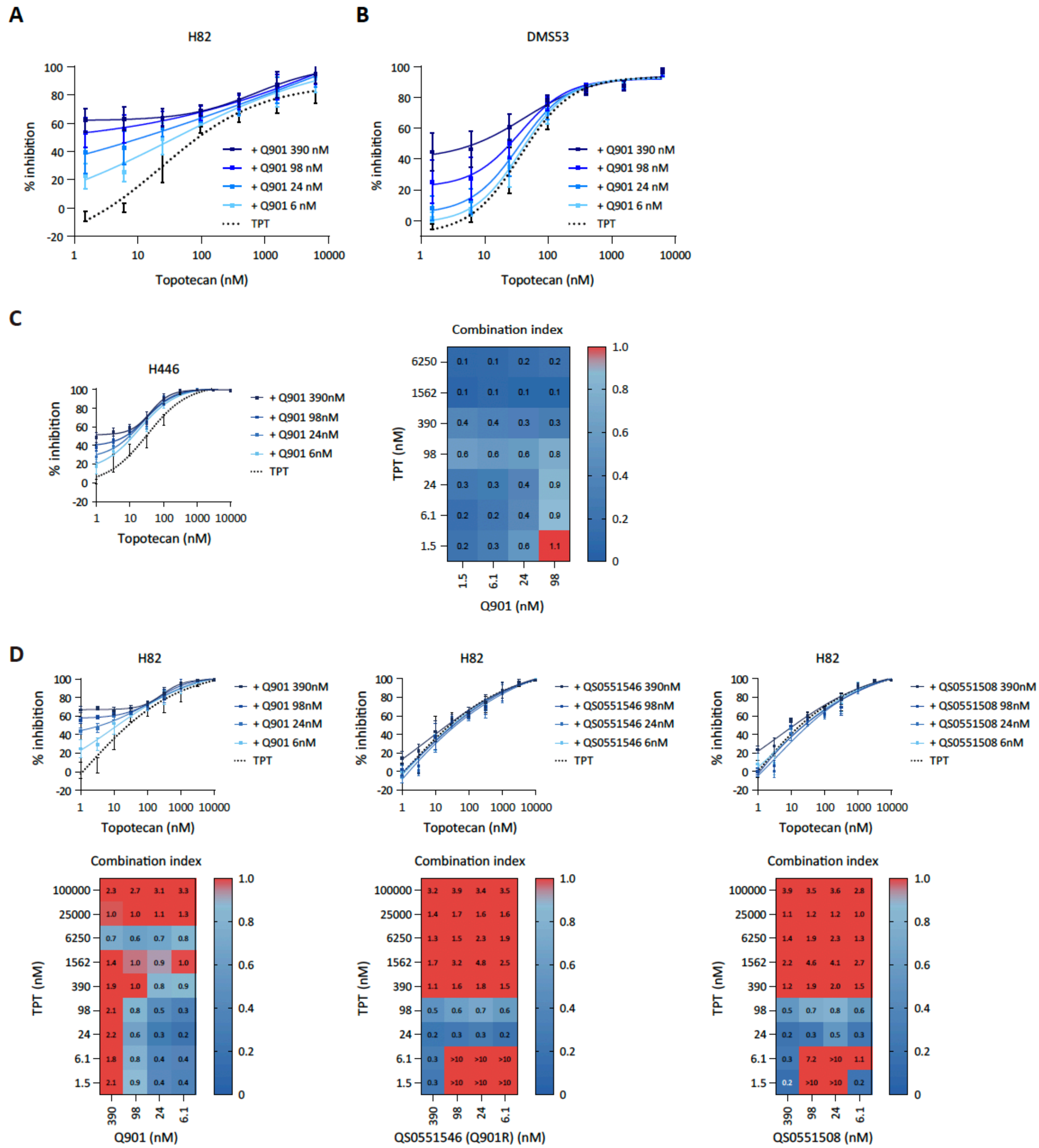

Fig. S13. Enhanced effect of Q901 in combination with a TOP1 inhibitor.
(**A** and **B**) Human SCLC cells, H82 (A), and DMS53 (B), were treated with varying concentrations of Q901 in combination with TPT for 72 h. Cell viability was measured using the CellTiter-Glo assay system. The percentage of cell growth inhibition was quantified by normalizing luminescence readings to untreated controls (n = 2, data represent mean ± SD). (**C**) CTG assay and combination index heatmap of H446 cell line. (**D**) CTG assay and combination index heatmap of three CDK7 inhibitors in combination with TPT.

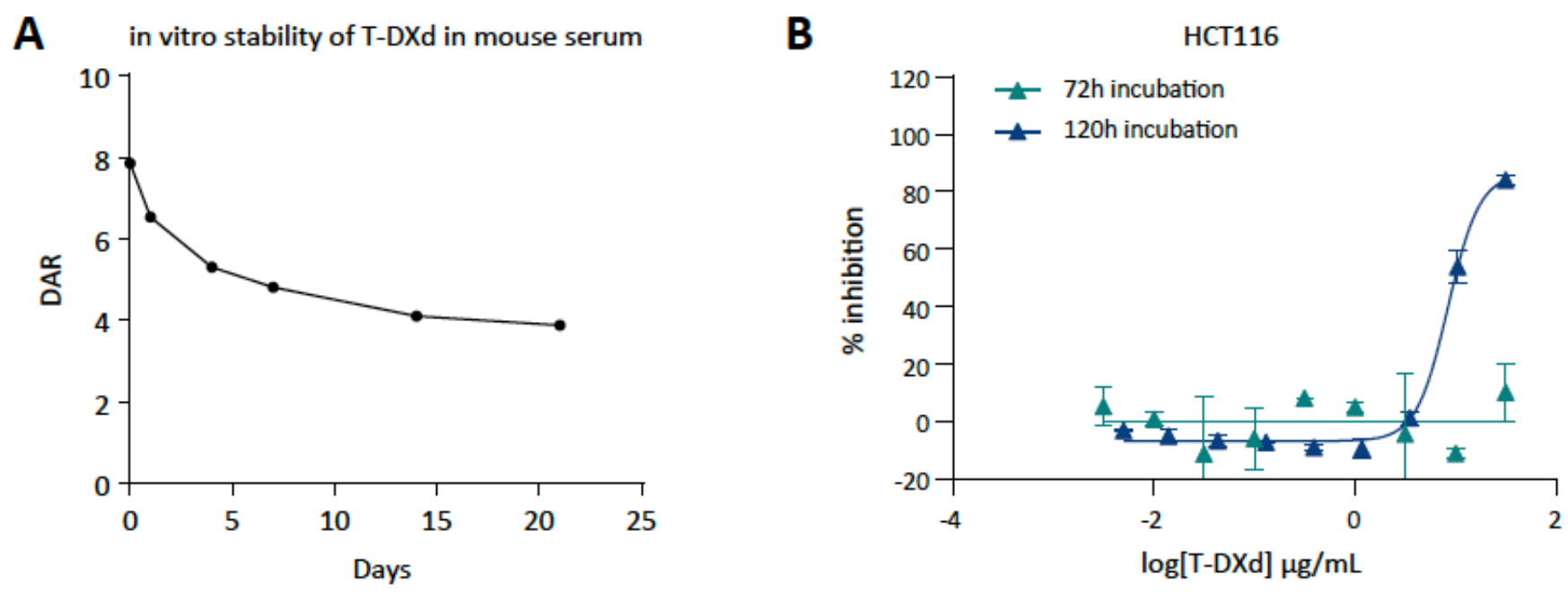

Fig. S14. Serum stability and DAR profiling of T-DXd over time. Serum stability and DAR profiling of T-DXd over time.
(**A**) A gradual shift in the DAR of T-DXd from 8 to 4 was observed by day 21, indicating sustained payload release in mouse serum. (**B**) Time-dependent cellular response to T-DXd in HCT116 cells measured using a CTG assay. Cells were treated with T-DXd across a 10-point concentration range (0–31.6 µg/mL, 1:3.16 serial dilution) for 72 h or 120 h. Data are presented as % inhibition relative to control.

CDKs	Residual activity at 1μM (% of control)	IC_{50}, (μM)
CDK7/CycH/MAT1	1	0.01
CDK1/CycA2	90	8.71
CDK1/CycB1	93	>10
CDK1/CycE1	92	5.00
CDK12/CycK	84	2.48
CDK13/CycK	N/A	7.69
CDK16/CycY	95	6.94
CDK19/CycC	105	>10
CDK2/CycA2	91	>10
CDK2/CycE1	97	8.09
CDK3/CycC	85	>10
CDK3/CycE1	111	>10
CDK4/CycD1	96	>10
CDK4/CycD3	91	>10
CDK5/p25NCK	93	>10
CDK5/p35NCK	93	>10
CDK6/CycD1	96	>10
CDK6/CycD3	100	>10
CDK8/CycC	86	>10
CDK9/CycK	86	8.58
CDK9/CycT1	91	>10

Table S1. The selectivity of Q901 against different CDK/Cyclin pairs.
N/A: Not Applicable.
